# The Red Queen Model of Recombination Hotspots Evolution in the Light of Archaic and Modern Human Genomes

**DOI:** 10.1371/journal.pgen.1004790

**Published:** 2014-11-13

**Authors:** Yann Lesecque, Sylvain Glémin, Nicolas Lartillot, Dominique Mouchiroud, Laurent Duret

**Affiliations:** 1 Laboratoire de Biométrie et Biologie Evolutive, UMR CNRS 5558, Université Lyon 1, Villeurbanne, France; 2 Institut des Sciences de l'Evolution, UMR CNRS 5554, Université Montpellier 2, Montpellier, France; University of California Davis, United States of America

## Abstract

Recombination is an essential process in eukaryotes, which increases diversity by disrupting genetic linkage between loci and ensures the proper segregation of chromosomes during meiosis. In the human genome, recombination events are clustered in hotspots, whose location is determined by the PRDM9 protein. There is evidence that the location of hotspots evolves rapidly, as a consequence of changes in PRDM9 DNA-binding domain. However, the reasons for these changes and the rate at which they occur are not known. In this study, we investigated the evolution of human hotspot loci and of PRDM9 target motifs, both in modern and archaic human lineages (Denisovan) to quantify the dynamic of hotspot turnover during the recent period of human evolution. We show that present-day human hotspots are young: they have been active only during the last 10% of the time since the divergence from chimpanzee, starting to be operating shortly before the split between Denisovans and modern humans. Surprisingly, however, our analyses indicate that Denisovan recombination hotspots did not overlap with modern human ones, despite sharing similar PRDM9 target motifs. We further show that high-affinity PRDM9 target motifs are subject to a strong self-destructive drive, known as biased gene conversion (BGC), which should lead to the loss of the majority of them in the next 3 MYR. This depletion of PRDM9 genomic targets is expected to decrease fitness, and thereby to favor new PRDM9 alleles binding different motifs. Our refined estimates of the age and life expectancy of human hotspots provide empirical evidence in support of the Red Queen hypothesis of recombination hotspots evolution.

## Author Summary

In eukaryotic genomes, recombination plays a central role by ensuring the proper segregation of chromosomes during meiosis and increasing genetic diversity at the population scale. Recombination events are not uniformly distributed along chromosomes, but cluster in narrow regions called hotspots. The absence of overlap between human and chimpanzee hotspots indicates that the location of these hotspots evolves rapidly. However, the reasons for this rapid dynamic are still unknown. To gain insight into the processes driving the evolution of recombination hotspots we analyzed the recent history of human hotspots, using the genome of a closely related archaic hominid, Denisovan. We searched for genomic signatures of past recombination activity and compared them to present-day patterns of recombination in humans. Our results show that human hotspots are younger than previously thought and that they are not conserved in Denisovans. Moreover, we confirm that hotspots are subject to a self-destruction process, due to biased gene conversion. We quantified this process, and showed that its intensity is strong enough to cause the fast turnover of human hotspots.

## Introduction

Meiotic recombination is a highly regulated process, initiated by the programmed formation of double-strand breaks (DSBs). These DSBs are subsequently repaired, using homologous chromosomes as a template, thus leading to crossover (CO) or non-crossover (NCO) recombination events. In mammals, as in many other eukaryotes, the formation of at least one CO on each chromosome is required for the proper disjunction of chromosomes during meiosis (for review see [1]). Hence, the recombination machinery must be tightly controlled to promote a sufficient number of COs on each chromosome, while ensuring that all DSBs can be efficiently repaired to produce viable gametes.

Recombination events are not randomly distributed across the genome, but cluster in hotspots, typically 1 to 2 kb long [2]–[8]. About 33,000 recombination hotspots have been identified in the human genome, which account for 60% of COs and 6% of the sequence [2]. Many independent observations have clearly demonstrated that in human and mouse, the location of hotspots is primarily determined by the zinc finger protein PRDM9, through its sequence-specific DNA-binding domain [9]–[12]. PRDM9 contains a SET domain, which catalyzes histone H3 Lys4 trimethylation (H3K4me3) at hotspot loci [4], [12]–[14]. PRDM9 is highly polymorphic, specifically in its DNA binding domain, and the location of recombination hotspots differs among individuals carrying different alleles [9], [11], [12], [15]. At the population scale, the set of recombination hotspots that are the most frequently used can be inferred from patterns of linkage disequilibrium [3] or of genetic admixture [16]. These analyses revealed that more than 90% of recombination hotspots are shared between European and African populations [16]. This strong overlap is due to the fact that the same major allele of PRDM9 (allele A) is present at high frequency both in European and African populations [11]. Interestingly, this A allele presents affinity for the 13-bp motif *CCTCCCTNNCCAC*, which was initially identified on the basis of its enrichment within human recombination hotspots [17] (we will hereafter refer to this sequence motif as HM – for human hotspot motif).

It has been shown that the location of recombination hotspots is not conserved between human and chimpanzee [18]–[20]. This rapid shift is presumed to be due to the fact that the major PRDM9 alleles present in each species have different DNA binding specificities [10], [19]. There is clear evidence that PRDM9 has evolved under strong positive selection, in primates as well as in many other animal lineages, specifically at those sites involved in DNA sequence recognition [21], [22]. This indicates that PRDM9 has been under selective pressure to switch to new targets [21], [22]. However, the reasons for this selective pressure remain mysterious.

One interesting hypothesis, proposed by Myers and colleagues [10], is that the turnover of PRDM9 alleles might be a consequence of the self-destruction of recombination hotspots by the process of biased gene conversion (BGC) [23]–[25]. Indeed, the repair of DSBs is expected to lead to the conversion of recombination-prone alleles by hotspot-disrupting alleles [23]–[25] (we will hereafter refer to this form of BGC as 'dBGC', for DSB-driven BGC). In agreement with the dBGC model, it was shown that the HM motif was subject to accelerated evolution in the human lineage [10]. The authors suggested that the progressive degradation of recombination hotspots through dBGC might lead to a loss of fitness. Indeed, there is evidence that lower CO rates are associated with lower fertility, possibly due to improper chromosome disjunction [26]. Hence, the loss of PRDM9 target motifs might favor the increase in frequency of new PRDM9 alleles, targeting different motifs [10].

Simulation studies have shown that this model, termed the 'red queen theory of recombination hotspots', might explain the rapid turnover of recombination hotspots [27]. It is however not established whether this model is quantitatively realistic. Notably it has been argued that the number of human recombination hotspots (∼30,000) largely exceeds the number of COs per meiosis (∼60) and hence is unlikely to be limiting [22]. The comparison of human and chimpanzee PRDM9 genes revealed multiple non-synonymous changes driven by positive selection in each lineage [22]. If the red queen model is correct, this implies that this turnover process (loss of PRDM9 targets by dBGC leading to a selective pressure that favored new PRDM9 allele) occurred several times since the divergence between human and chimpanzee. Thus, one strong prediction of the red queen model is that the life expectancy of PRDM9 target motifs should be much shorter than the human/chimpanzee divergence time. In other words, the key issue is to determine whether, during the lifespan of a given PRDM9 allele, the loss of its target motifs by dBGC is fast enough to have a significant impact on genome-wide recombination patterns. To address this issue, we first determined when the HM motif started to be the target of PRDM9 allele A in the human lineage. For this, we analyzed the genome sequence of a Denisovan individual [28], an archaic human that diverged from the modern humans about 400,000–800,000 years ago [29]. We then used polymorphism data to quantify the strength of dBGC on HM motifs in extant human populations. This combined analysis of polymorphism and divergence, made possible thanks to Denisovan genomic data, demonstrates that the life expectancy of human recombination hotspots is very short, and brings support for the red queen theory of recombination hotspots.

## Results

### The HM motif started to be targeted by PRMD9 shortly before the Human-Denisovan split

**Figure 1:**
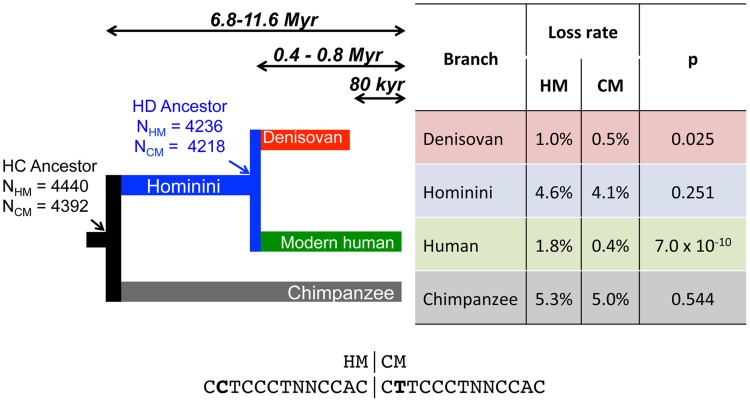
Differential loss of HM motifs across recent human history. The number of intact HM and CM motifs found in the reconstructed sequence (F2 subset) of human and chimpanzee last common ancestor (HC) and in the last human-Denisovan common ancestor (HD) is indicated with a simple arrow. Loss rates of HM and CM motifs are indicated for each branch. HM and CM loss rates were compared with a proportion test (p-value: *p*). Sequences of both motifs are shown below the tree. Double arrows represent populations divergence times [28], [29].

The major human allele of PRDM9 (allele A, present at a frequency of 84% in European populations and 50% in African populations [11]) recognizes a specific sequence motif, whose core consensus is *CCTCCCTNNCCAC* [9], [10]. This motif promotes recombination specifically in humans, not in chimpanzee, and is particularly active in the context of THE1 transposable elements [10], [19]. As predicted by the self-destructive dBGC drive model, it was previously shown that this motif has accumulated an excess of substitutions specifically in the human lineage, after its divergence from chimpanzee, and that the HM loss rate was particularly strong within THE1 elements [10]. Based on the dBGC model [25], the authors proposed that HM had been active for a period of time corresponding to the last 20% to 40% of the time since the human-chimpanzee split [10]. This estimate was however based on poorly known parameters, and was therefore provided as a conservative upper bound [10]. To obtain a more direct dating of the onset of the HM motif activity, we used the Denisovan genome so as to determine when the HM motifs started to be subject to dBGC during the evolution of modern and archaic humans (Figure 1). We analyzed the evolution of HM motifs both within and outside human recombination hotspots. For this, we used recombination maps inferred by HapMap from patterns of linkage disequilibrium in human populations [2]. These maps reflect the average crossover rates across human populations over many generations. We will hereafter refer to these data as human "historical" recombination rates. Given that the list of human historical hotspots is currently available only for autosomes, we excluded sex chromosomes from our analyses.

**Figure 2:**
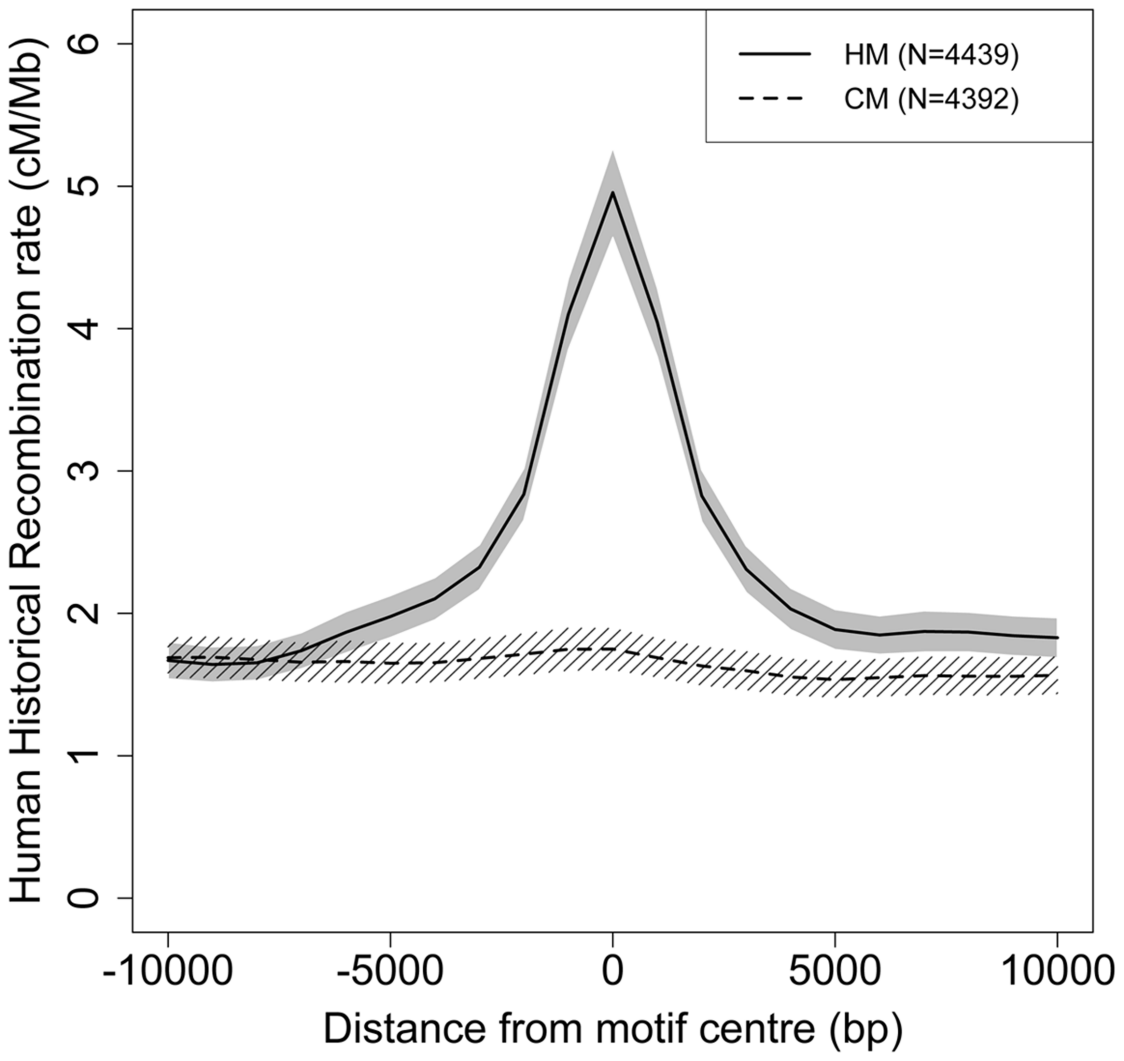
Modern human recombination profiles around HM and CM motifs found in the HC-ancestral sequence. Human historical recombination rates (cM/Mb) around CM (dotted line) and HM (solid line) motifs found in the human-chimpanzee reconstructed ancestral sequence (F2 subset). Recombination rates are averaged on 2 kb overlapping windows (overlap  = 1 kb). The 95% confidence interval of the mean recombination rate is shown by the grey area for HM and the hatched area for CM.

We first identified HM motifs (N = 5,704) in the reconstructed autosomal sequences of the human-chimpanzee ancestor (HC), and then counted base replacement changes along the four branches of the phylogeny (hereafter termed modern human, Denisovan, Hominini and chimpanzee branches, Figure 1), by comparing sequences of reference genomes to the ancestral one (see methods). It should be noted that the detected base changes include both fixed and polymorphic mutations. To quantify the excess of base changes (if any) on HM motifs along each branch of the phylogeny, we used as a reference the rate of base change within a control motif (CM: *CTTCCCTNNCCAC, N = 5,483*), which differs from HM by the second position and does not show any effect on the recombination pattern [30] (Figure 2).

We counted base changes only at informative sites (i.e. we ignored the two N positions) and excluded the second position, which differs between HM and CM motifs. Thus, we only examined sites that are *a priori* expected to have the same rate of mutation (and possibly sequencing errors) in HM and CM motifs. We considered a motif to be lost as soon as it was subject to one mutation in one informative site. To minimize errors in the inference of motif losses, it is necessary to avoid regions with low sequencing quality or erroneous alignment. Thus, we created three levels of filters (F1, F2 and F3) successively applied to our data so as to keep three subsets of motifs. A motif is discarded from a subset if at least one informative site does not pass the filter. Filter F1 retains all aligned sites common to human, chimpanzee and Denisovan, while filter F2 favors a more accurate HC ancestral sequence reconstruction. Finally, the most stringent filter F3 accounts for sequence errors specific to ancient DNA in Denisovan (see methods). Unless explicitly mentioned, results presented below correspond to the F2 dataset, totalizing 4,440 HM and 4,393 CM motifs present in the human-chimpanzee ancestor.

In the modern human branch, we observed that the HM loss rate (1.8%) is more than four times higher than the CM loss rate (0.4%; green branch in Figure 1). As expected, the HM loss rate is much higher within THE1 elements (6.7% vs. 1.7%; proportion test: p = 8.2×10^−5^) (Table 1). However, the excess of HM losses is not limited to THE1 elements: at non-THE1 loci, the HM loss rate is significantly higher than the CM loss rate (1.7% vs. 0.4%, p = 2×10^−8^). Conversely, we observed no significant difference in HM and CM loss rates along the Chimpanzee branch (in grey in Figure 1), as expected given that the HM motif is not a target of PRDM9 alleles in chimpanzees [10], [19]. This negative control confirms that there is no intrinsic difference in mutation rate between the two motifs, and hence that the CM motif is a good reference to detect accelerated evolution of the HM motif.

**Table 1:** HM motifs loss rates within *versus* outside THE1 elements.

Branch	Within THE1		Outside THE1		p^c^
	N^a^	Rate^b^	N^a^	Rate^b^	
Chimpanzee	145	5.5%	4295	5.3%	0.999
Hominini	145	7.6%	4295	4.5%	0.122
Denisovan	134	0.0%	4102	1.0%	0.463
Human	134	6.7%	4102	1.7%	8.2×10^−5^

a Intact motif count at ancestral edge of the branch (cf. Figure 1).

b Motif loss rate along the branch.

c P-value of proportion test comparing HM loss rates within *vs.* outside THE1 elements along the branch.

**Figure 3:**
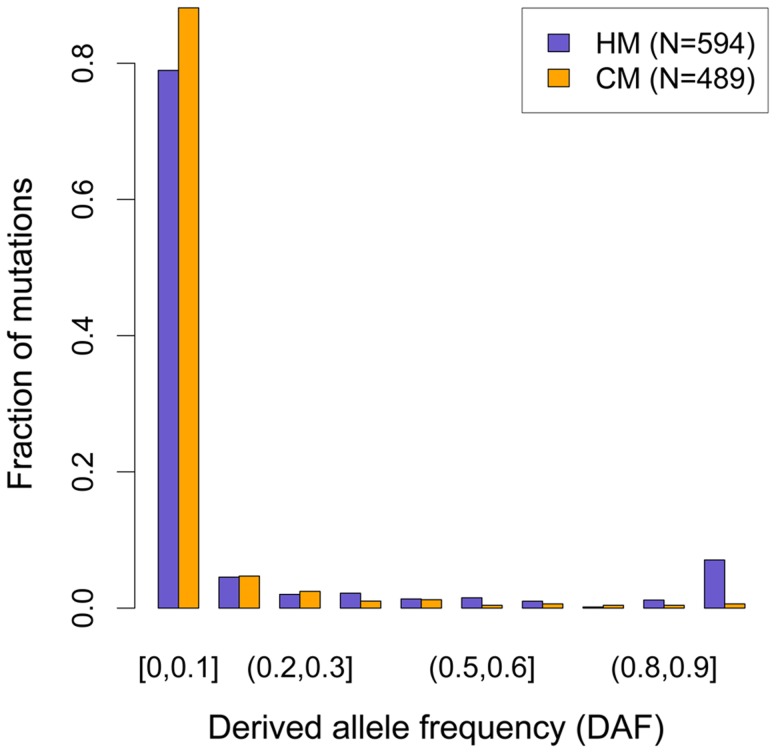
Derived allele frequency (DAF) spectra of mutations leading to motifs loss in the human branch. DAF of mutations affecting HM (purple bars) and CM (orange bars) along the human branch (green branch in Figure 1). Allele frequencies are extracted from 1000 genomes phase I, using all available populations [31]. Mutations count for each motif (F2 subset) is indicated (N).

These observations are consistent with the self-destructive dBGC drive model. However, they could also be explained by a possible mutagenic effect of recombination. To distinguish between these two possibilities, we analyzed the derived allele frequency (DAF) spectra of mutations in HM and CM motifs: under the hypothesis that the increased HM loss rate is simply due to a higher mutation rate (and not a fixation bias, like BGC), the two DAF spectra are expected to be identical. We included in these analyses all modern-human mutations detected as polymorphic by the 1000 genomes project [31], as well as fixed ones. We observed that the DAF spectrum of HM mutations is shifted towards higher frequencies compared to CM mutations (Figure 3), with an average mean DAF almost three times higher (13% vs. 5%; Wilcoxon test p = 1.9×10^−6^). Overall 3.7% of HM mutations detected in the modern human branch are fixed, compared to 0.2% for CM mutations (Proportion test p = 1.6×10^−4^). This demonstrates that the accumulation of HM losses in the human branch is a consequence of a fixation bias, as predicted by the dBGC model [32].

In chimpanzee, HM is not a target of PRDM9 and hence is not expected to be subject to any fixation bias. Consistent with this prediction, the mean DAF of HM mutations in chimpanzee (0.28) is not higher than that of CM mutations (0.35), and overall there is no significant difference in the DAF spectra of these two categories of mutations (Figure S1). We note however that, given the small sample size (66 and 67 polymorphic mutations in HM and CM motifs, respectively), the power to detect fixations biases is lower in chimpanzees than in humans.

In the Hominini branch, ancestral to Denisovans and modern humans (in blue in Figure 1), the HM loss rate appears slightly higher than the CM loss rate, but this difference is not statistically significant. In the Denisovan branch (in red in Figure 1), the HM loss rate (1%) is two times higher than the CM loss rate (0.5%). This excess is weaker than that observed in the modern human branch, but it is still significant (p = 0.025). Additionally, the rate of homozygosity of these mutations (computed using the diploid sequence of the Denisovan individual) is higher for HM than for CM (0.80 *vs.* 0.69). This trend is consistent with the hypothesis that in Denisovans, as in modern humans, HM mutations segregated on average at higher frequency than CM mutations. The fact that the signature of dBGC on HM motifs is weaker in Denisovan compared to human might be explained by slightly different sequence affinities of their PRDM9 alleles, or by a lower population frequency of HM-targeting PRDM9 alleles in Denisovans. This weaker signature of dBGC might also be due to the fact that the effective population size was smaller in Denisovans compared to modern humans [33], which is expected to enhance the effects of random genetic drift, and hence to decrease the strength of dBGC [32].

Given that ancient DNA is prone to sequencing errors, we repeated our analyses with more stringent criteria to keep only data with the highest sequence quality (filter F3). In that F3 subset, we found the same two-fold excess of HM losses compared to CM losses in the Denisovan branch (Table S1). Overall, the three filters (F1, F2 or F3) lead to the same conclusion: there is a strong signal of dBGC on HM motifs in the terminal branches (stronger in humans than in Denisovans), and a weak signal of dBGC in the Hominini branch (Table S1, S2 and Figure 1). In each of these three branches, the observed excess of HM losses relative to CM losses is the strongest with the most stringent F3 filter (Table S1, S2). However, given that the power of statistical tests decreases as the sample size decreases, the slight excess of HM losses in the Hominini branch is detected as statistically significant only in the F1 dataset (Table S2), and the signal of dBGC in the Denisovan branch becomes non-significant in the F3 dataset (Table S1). All these observations indicate that HM has been subject to dBGC both in Denisovans and modern humans lineages, and suggest that HM started to be a target of PRDM9 shortly before the Denisovan/modern human split.

### Quantifying the intensity of dBGC on HM motifs in the human branch

Given that the CM motif is not recombigenic (Figure 2), the shift in DAF spectra observed between CM and HM mutations (Figure 3), can entirely be attributed to dBGC acting on HM motifs. To estimate the intensity of dBGC against HM motifs, we fitted a population genetic model to the DAF spectra of CM and HM mutations, considering CM mutations as neutral references (see methods). Since dBGC behaves like selection on semi-dominant mutations [32], we used the model of Eyre-Walker et al. [34] to quantify it. Under the simplifying assumption that all HM informative sites are subject to the same dBGC strength, the population scaled dBGC coefficient (*G* = 4*N_e_g*) estimated on all HM motifs is 8.55 (95% confidence interval  = 2.76–2655). This result is robust to the number of categories used to describe DAF spectra (Table S3). It should be noticed that large values of G are difficult to estimate accurately because above a given threshold (G>20), all values of this parameter are expected to give very similar DAF spectra (Figure S2). This explains why the upper bound of the confidence interval of this estimate of G is very high.

**Figure 4:**
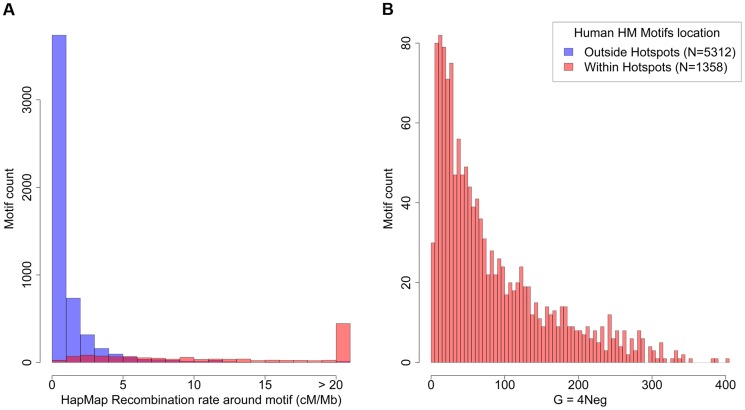
Recombination rates and strength of dBGC on HM motifs in the human genome. (A) Distribution of human historical recombination rates (cM/Mb), measured over a 2 kb window centered on HM motifs from human autosomes (hg19 assembly; no filter). Red: motifs located within historical recombination hotspots. Blue: motifs located outside hotspots. (B) Distribution of estimated population-scaled dBGC coefficient (*G*) on HM motifs located in recombination hotspots in the human genome. Median  = 57.5.

The strength of dBGC at a given locus is proportional to the absolute difference in recombination rate between the original (hot) allele and the mutant (colder) allele [25]. This difference can be large only if the recombination rate at this locus is high. Hence HM motifs that are located at lowly recombining loci are not expected to undergo dBGC. It is important to note that the recombination rate at HM motifs is highly variable across the genome: 8% of HM motifs concentrate 60% of all crossover events located in the vicinity of HM motifs (±2 kb) (Figure 4A). It is therefore expected that the intensity of dBGC should be stronger for HM motifs located in a genomic context prone to recombination. To test that prediction, we re-estimated *G* from DAF spectra, in three subsets of equal sample size, binned according to the local historical recombination rate (measured on a 2 kb window centered on motif position). As expected, *G* increases with increasing historical recombination rates, from *G* = 0.96 in the first tercile to *G* = 14.64 in the third tercile (Table S4).

To get a better picture of the distribution of *G* across all HM motifs, we fitted a simple model where the dBGC coefficient at a given locus is directly proportional to the local crossover rate at this locus (Text S1). The distribution of G inferred by the model (given the observed distribution of recombination rates around HM motifs), indicates a median value of G = 57.5 for HM motifs located within historical hotspots (Figure 4). The 8% most highly recombining motifs are predicted to be subject to very strong dBGC (on average, G = 174, CI: 29–291).

### Mutations of HM motifs do not immediately silence recombination hotspots

**Figure 5:**
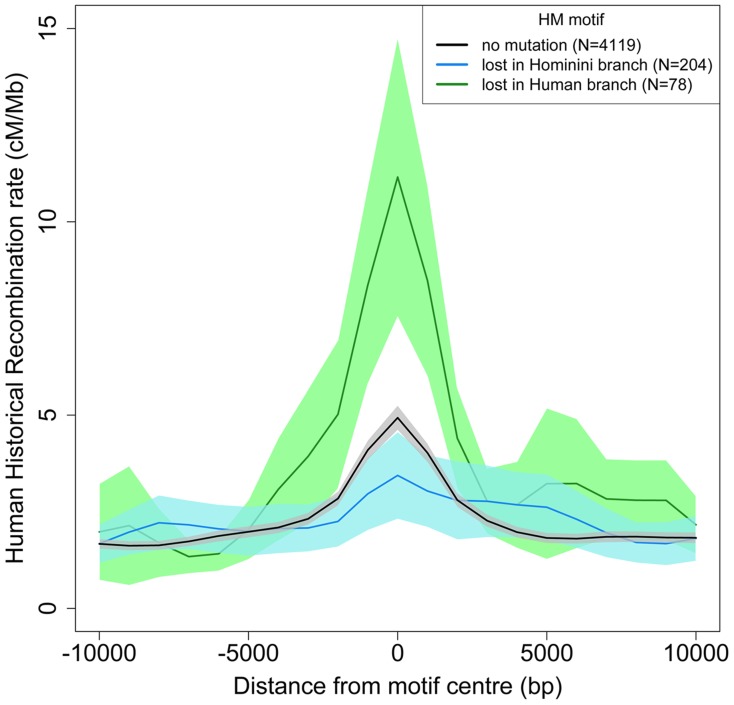
Historical human recombination profiles around lost and conserved HM motifs. Human historical recombination rates (cM/Mb) around HM motifs found in the human-Chimpanzee reconstructed ancestral sequence (F2 subset) and conserved in the human genome (black) or lost in the Hominini (blue) or human (green) branch. The 95% confidence interval of the mean recombination rates is shown by areas colored accordingly. Recombination rates are averaged on 2 kb overlapping windows (overlap  = 1 kb).

The dBGC model predicts that the small subset of HM motifs located in a highly recombining context should accumulate substitutions extremely rapidly. In agreement with that prediction, we observed that, along the modern human branch, the loss rate is almost 3 times higher for HM motifs located within historical hotspots compared to other HM motifs (3.5% *vs.* 1.2%; p = 4.6×10^−7^). Overall, 55% of the HM motifs detected as being mutated along the modern human branch are located within historical recombination hotspots (compared to 28% for motifs that have remained intact) (Table 2). Thus, on average, the historical recombination rate at HM motifs mutated in the modern human branch is more than two times higher than that at intact HM motifs (11.2 cM/Mb vs. 4.9 cM/Mb; Figure 5). Notably, we observed the same pattern with present-day recombination rates, inferred from pedigree-based genetic maps [35] (Figure S3). Moreover this pattern is observed even for the subset of HM mutations that are fixed in human populations (Figure S4). These observations show that mutations of HM motifs that were fixed in modern humans are generally located in loci that still have a high recombination activity in present-day populations. Hence, although mutations of HM motifs diminish the local recombination rate, they generally do not directly convert a hotspot into a coldspot.

**Table 2:** Motifs loss rates within and outside modern human recombination hotspots (HapMap).

Motifs	Branch	N^a^		Rate^b^		p^c^	# obs. loss^d^	# exp. loss^e^	# dBGC loss^f^
		HM	CM	HM	CM				
Within hotspots	Chimpanzee	1272	724	5.5%	5.4%	0.994			
	Hominini	1272	724	3.9%	4.3%	0.792			
	Denisovan	1222	693	0.8%	0.4%	0.485			
	Human	1222	693	3.5%	0.6%	1.2×10^−4^	43	7	36
Outside hotspots	Chimpanzee	3167	3669	5.2%	4.9%	0.605			
	Hominini	3167	3669	4.9%	4.0%	0.109			
	Denisovan	3013	3521	1.1%	0.5%	0.024	32	15	17
	Human	3013	3521	1.2%	0.4%	3.3×10^−4^	35	12	23

a Intact motif count at ancestral node of the branch (cf. Figure 1).

b Motif loss rate along the branch.

c P-value of proportion test comparing HM *vs.* CM loss rates along the branch.

d Number of observed HM losses.

e Number of HM losses expected in absence of dBGC (i.e. based on CM loss rate).

f Estimated number of HM losses caused by dBGC (obs. - exp.). NB: these values are reported only for lineages showing evidence of dBGC (p<0.05).

Interestingly, HM motifs that are located outside of historical recombination hotspots also show a signature of dBGC. This signature is weaker than for HM located within hotspots, but still clearly significant: there is a 3-fold excess of HM losses compared to CM losses in the modern human branch (Table 2), and HM mutations segregate at higher frequencies than CM mutations (10% *vs.* 4%; p = 0.0014). The analysis of present-day recombination rates confirmed the absence of recombination hotspots at these mutated HM sites (Figure S5). This suggests that these HM losses occurred in ancient recombination hotspots that are not active anymore. Overall, we detected 78 HM losses along the modern human branch, whereas only 19 would have been expected if the loss rate were the same as that of CM motifs. Among these 59 extra losses that can be attributed to dBGC, 23 occurred at loci that are not detected as recombination hotspots (Table 2). Thus, among all loci that used to be recombination hotspots in the human lineage and that have lost the HM motif by dBGC, 39% are no longer active.

### No overlap between human and Denisovan recombination hotspots

With only one single individual sequenced, it is not possible to establish recombination maps in Denisovans. However, different analyses can be performed to test whether recombination hotspots identified in modern human populations correspond to hotspots in Denisovans.

**Figure 6:**
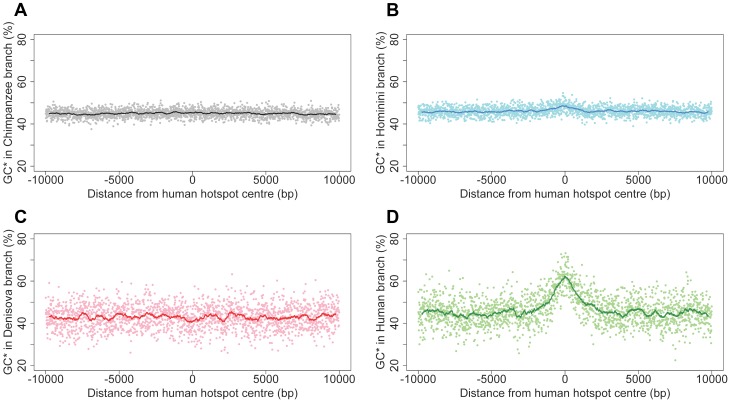
Equilibrium GC-content (GC*) around human recombination hotspots in different branches of the phylogeny. GC* is computed on each branch of the phylogeny (Figure 1): (A) Chimpanzee branch; (B) Hominini branch; (C) Denisovan branch; (D) Modern human branch. Profiles show the mean GC* computed on 32,987 human historical hotspots, over a 20 kb region centered on the middle of hotspots. Each dot is the average GC* over a 10 bp window. The line shows average GC* over 500 bp window.

A first approach to detect past recombination activity consists in analyzing substitution patterns, so as to infer the equilibrium GC-content (denoted GC*) along different branches of the phylogeny (see methods). Many lines of evidence indicate that in primates, recombination is driving the evolution of GC-content via the process of GC-biased gene conversion (gBGC), which results from a bias in the repair of AT:GC mismatches in heteroduplex DNA during meiotic recombination [36], [37]. Notably, it has been shown that GC* strongly correlates with present or past recombination rates [38]–[40]. We therefore measured GC* separately for each branch of the phylogeny at loci corresponding to the 32,981 human historical recombination hotspots [2]. As expected, we observed a strong peak of GC* centered on the middle of historical recombination hotspots, in the modern human branch (Figure 6D). In agreement with previous results [19], this peak is absent in the chimpanzee branch (Figure 6A), consistent with the fact that human and chimpanzee recombination hotspots do not overlap. Interestingly, we observed only a very limited bump of GC* in the Hominini branch (Figure 6B). This indicates that, up to a recent time, shortly before the Denisovan/modern human split, loci corresponding to human historical recombination hotspots were not subject to gBGC.

Surprisingly, we observed no peak of GC* in the Denisovan branch (Figure 6C). This result was unexpected: given our observations indicating that the HM motif started to be a target of PRDM9 before the split between modern humans and Denisovans, we presumed, *a priori*, that the two populations should share the same recombination hotspots. We first hypothesized that the absence of peak of GC* could be due to the fact that, owing to the relatively low effective population size in Denisovan, gBGC was too weak to leave any detectable signature. To test this hypothesis, we investigated whether we could detect the hallmarks of gBGC in Denisovan, by analyzing correlations between GC* (inferred along different branches of the phylogeny) and recombination rates, measured in 1 Mb-windows. At this genomic scale, recombination rates are well conserved between human and chimpanzee [19] and hence are expected to be also conserved in Denisovan. As predicted by the gBGC model, and in agreement with previous results [38]–[40], we observed a significant correlation between human historical recombination rates and GC* along the modern human branch (R^2^ = 13%; p<10^−74^). This correlation is as strong for GC* computed in the Denisovan branch (R^2^ = 14%; p<10^−80^; Figure S6), which indicates that, genome-wide, the signature of gBGC is as visible in Denisovan as it is in human. Thus, the absence of peak of GC* in the Denisovan branch at human recombination hotspots loci cannot be attributed to a possibly weaker gBGC effect in Denisovan. Instead, it indicates that recombination hotspots were not shared between humans and Denisovans.

To further test this conclusion, we used an independent approach. The self-destructive drive model predicts that HM motifs located in recombination hotspots should be subject to stronger dBGC than other HM motifs. Thus, if the location of recombination hotspots was conserved, then HM motifs located in loci corresponding to human recombination hotspots should show an enhanced signature of dBGC not only in human (as shown previously), but also in the Denisovan branch. As already mentioned, we observed an excess of HM losses compared to CM losses in Denisovan (Figure 1), which indicates that there is a detectable signature of dBGC on HM in Denisovan. However, the HM loss rate is not different between HM loci that correspond to human historical hotspots and other HM loci (respectively 0.8% and 1.1%, p = 0.579; Table 2). Thus, we see no evidence for stronger dBGC in Denisovan at the location of human historical hotspots.

Finally, it has been shown that HM motifs located within THE1 transposable elements are particularly prone to recombination in humans [10], [17]. As expected, we observed a markedly elevated HM loss rate within THE1 elements in human (6.7%). In contrast, we did not detect any mutation in the Denisovan branch among HM motifs located in THE1 (loss rate  = 0%; proportion test: p = 0.0067; Table 1). This suggests that contrarily to human, HM motifs located within THE1 elements were not associated to elevated recombination rates in Denisovan.

All these observations concur to the conclusion that fine-scale recombination rates were not conserved between Denisovans and humans. This therefore suggests that the A allele of PRDM9 was either absent or present at very low frequency in Denisovans. The major PRDM9 allele in Denisovans was probably similar to the A allele (since it also had affinity for the HM motif), but it targeted recombination hotspots to a different subset of HM motifs.

### Expected lifespan of human recombination hotspots

The lifetime of HM motifs can be predicted using standard population genetic approximation [25]. Under the simplifying assumption that motif mutations are immediately either lost or fixed in the population, the probability that a hotspot motif accumulates at least one disrupting substitution after *T* generations, can be approximated by:
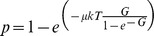
where *µ* is the mutation rate (1.2×10^−8^ mutations/bp/generation in humans [41]), *k* the length of the motif (here *k* = 11 for the HM motif) and *G* the population-scaled dBGC coefficient (G = 4N_e_g) [25].

Given the distribution of G estimated previously, this model predicts that after 100,000 generations (i.e. about 3 MYR [29]), overall, 18% of HM motifs should be lost (CI: 5%–23%). But importantly, for the subset of most highly-recombining HM motifs (top 8% of HM motifs, which concentrate 60% of HM-associated recombination events), the predicted loss rate is extremely high (87%; CI: 32%–96%). Thus the model indicates that if the dBGC drive against HM remains as strong as it is in extant human populations, then the subset of highly-recombining HM motifs should be rapidly lost. We observed that in many cases, the loss of HM does not totally abolish the hotspot activity. This is most probably due to the fact that the affinity of PRDM9 depends not only on the HM motif, but also on interactions with other sites in its vicinity. However, our observations indicate that losses of a HM motif by dBGC in the human branch were associated with hotspot extinction in 39% of cases. Given that the human branch is relatively short (14,000–28,000 generations), this suggests that within the next 100,000 generations, the loss of HM motifs should be accompanied by the loss of recombination hotspots activity.

## Discussion

PRDM9 is the major determinant of the location of recombination hotspots in humans and mice [9]–[12], [15], [16], [35], [42]. At the population scale, the chromosomal distribution of recombination events is therefore expected to depend on the allelic composition at the PRDM9 locus. The location of human historical recombination hotspots reflects the DNA binding specificity of the A allele [9]. This allele is present at high frequency both in African and European populations, and as expected, most historical recombination hotspots are shared between these populations [16]. This implies that the majority of human historical recombination hotspots are older than 50,000 years. To determine more precisely the age of historical hotspots (i.e. to determine when the A allele started to reach substantial frequency within populations), we searched for signatures of recombination hotspot activity by analyzing patterns of sequence evolution across different branches of the phylogeny, before and after the divergence between modern humans and Denisovans. We used the fact that when a locus is recombining at a high rate (at the population scale), it then becomes subject to two forms of BGC: BGC in favor of mutations disrupting PRDM9 target motifs (dBGC), and BGC in favor of GC-alleles (gBGC).

Along the modern human branch, we observed clear signatures of dBGC against HM motifs: these motifs accumulated an excess of mutations, which tend to segregate at higher allelic frequencies. Moreover, we showed that the strength of this fixation bias in favor of HM-disrupting mutations increases with increasing local recombination rate. All these observations are perfectly consistent with the fact that HM is targeted by the major allele of PRDM9 in human populations (allele A). Interestingly, we also observed an excess of HM losses along the Denisovan branch, which suggests that HM started to be a target of PRDM9 before the population split between Denisovans and modern humans. However, several independent lines of evidence indicate that recombination hotspots were not shared between Denisovans and modern humans: in Denisovan, at loci corresponding to human recombination hotspots, we observed no signature of gBGC and no evidence of stronger dBGC against HM motifs. Moreover, in Denisovan, contrarily to human, HM motifs located within THE1 elements are not subject to accelerated loss.

The fact that fine-scale recombination rates were not conserved between humans and Denisovans might *a priori* seem in contradiction with the observation that the same motif (HM) was subject to accelerated loss in both lineages. However, the affinity of PRDM9 to its targets is not determined by this 13-bp motif alone, but also depends on interactions with surrounding sites [17], [43]. For example, in human, the HM motif is much more prone to recombination when located within the context of THE1 [17]. Overall, among the 6,671 HM motifs found in human autosomes, only 1,358 (20%) overlap with one of the 32,987 recombination hotspots identified by HapMap [2]. Thus, only a subset of HM motifs in the human genome are in a context for which the A allele of PRDM9 presents a high affinity. It is therefore possible that the major PRDM9 allele(s) present in Denisovan populations had affinity to HM, but within a different context.

In summary, the fact that Denisovans and humans had different hotspots but similar target motifs suggests that they had slightly different PRDM9 alleles, with distinct context specificity. This conclusion is compatible with two scenario: i) the A allele of PRDM9 was already present at a substantial frequency in the ancestral population (before the population split between Denisovans and modern humans), but was lost (or present at very low frequency) in the Denisovan lineage or ii) the A allele increased in frequency specifically in the modern human branch. Schwartz and colleagues [44] recently reported the partial genotyping of PRDM9 alleles present in the genomes of two archaic humans (the Denisovan genome analyzed here and that of an Altai Neandertal). In the Denisovan individual, they found evidence for the presence of an allele with a Zn-finger array composition different from that of the A allele, but compatible with rare PRDM9 alleles found in African populations [44]. By analyzing the copy number of Zn-finger repeat units, we further show that in fact the genotype of this Denisovan individual does not correspond to any known human PRDM9 allele (Text S2, Table S5, Figure S7, Dataset S1). Interestingly, the genome sequence of the Altai Neandertal individual also revealed the existence of another PRDM9 allele, different of the A allele [44]. However data from more individuals would be needed to determine whether or not the A allele was present in archaic human populations.

Myers and colleagues estimated that present-day human hotspots had been active for a period of time corresponding to at most 20% to 40% of the time since the human-chimpanzee split [10]. The analysis of substitution patterns along the different branches of the phylogeny allowed us to refine this estimate. At loci corresponding to human recombination hotspots, we observed a small bump of GC* in the Hominini branch (Figure 6B), which shows that some hotspots were already active in the human/Denisovan ancestor. Under the simplifying assumptions that all human recombination hotspots started to be active at the same date and that the intensity of gBGC has been constant since then, the onset of human hotspots activity can be dated to the last 10% of the time since the human-chimpanzee split (i.e. 0.7 to 1.3 MYR ago, depending on the estimate of the chimpanzee/human divergence time; see Text S3, Table S6, Table S7). Notably, the small excess of HM losses observed along the Hominini branch also indicates that the acceleration of HM loss rate may have started shortly before the human/Denisovan population split (see Text S3). Thus, the onset of activity of human historical hotspots coincides with the onset of dBGC on HM motifs. The most parsimonious explanation for these observations is that the A allele increased in frequency shortly before the human/Denisovan split.

To understand the dynamics of recombination hotspots it is necessary to establish not only when they were born, but also when they will die. The analysis of DAF spectra indicates that the subset of most highly-recombining HM motifs (top 8% of HM motifs, which concentrate 60% of HM-associated recombination events) is subject to very strong dBGC in extant human populations (G>90). If the intensity of dBGC remains stable over time, then after 100,000 generations (i.e. about 3 MYR), 87% (CI: 32%–96%) of these motifs are predicted to be lost. It should be noted that the DAF spectra of mutations in HM or CM motifs are very similar in African and European populations (Figure S8). Thus, despite the fact that the A allele is present at a higher frequency in European than in African populations (respectively 84% and 50% [11]), we see no evidence that the strength of dBGC against HM differs between the two populations. This might be due to the fact that the shift in PRDM9 allele frequency (and hence dBGC strength) arose recently, and did not have time to leave an imprint in DAF spectra of HM mutations. It is also possible that the effect of a higher frequency of the A allele in Europeans is mitigated by a stronger genetic drift in that population.

Up to now, the erosion of HM motifs in the modern human lineage has been quite limited: since the human/Denisovan split (14,000–28,000 generations), only 0.6% of HM motifs have accumulated fixed mutations (1.1% for motifs located within recombination hotspots). This relatively limited erosion can be explained by the fact that initially, when the A allele appeared and progressively increased in frequency in ancestral populations, the intensity of dBGC against HM motifs was certainly much weaker than it is in extant human populations (where the frequency of allele A reaches 50% to 90%). Moreover, many mutations did not have time to reach fixation. For instance, standard population genetics models [45] indicate that the fixation of a HM mutation subject to strong dBGC (G = 90) should take about 9,000 generations on average (for an effective population size in humans of 10,000). Thus, we are just observing the beginning of the erosion of HM motifs. However, in the long term (3 MYR), if the frequency of the A allele remains as high as in extant populations, the vast majority of the most active HM motifs are expected to be lost.

What might be the consequences of the genomic depletion of high affinity PRDM9 target sites? In mice, the knockout of *Prdm9* does not lead to a decrease the number of recombination hotspots [12]. However the location of hotspots in *Prdm9* ^-/-^ mice is totally different from that of wild-type mice, with a strong enrichment towards promoters and other sites of PRDM9-independent H3K4 trimethylation [12]. It is therefore plausible that the loss of high affinity PRDM9 target sites would also lead to relocate recombination hotspots to these regions. *Prdm9* ^-/-^ mice are sterile, which suggests that this re-patterning of recombination hotspot location is deleterious [12]. Hence, it is expected that the loss of high affinity PRDM9 target sites should provide a strong selective pressure for new PRDM9 alleles, with different DNA binding affinities, to rise in frequency in the population. This constraint is expected to appear much before all high affinity PRDM9 target sites have been lost. Thus, the next turnover of PRDM9 alleles (and hence of hotspot locations) is expected to occur before 3 MYR.

Our observations are therefore consistent with the Red Queen model of hotspot turnover [10], [27]. This does not imply that all cases of hotspot turnover are due to this evolutionary scenario: the mutation rate in the Zn-finger array is very high (due to the intrinsic instability of minisatellite repeats) and some new alleles may increase in frequency simply by random genetic drift. Notably the shift in hotspot location in Denisovans cannot be attributed to a shortage of HM motifs in the genome. This shift was more probably due to changes in PRDM9 allelic frequencies driven by random genetic drift in this small population. However, the Red Queen model provides a plausible and simple explanation for the recurrent selective pressure on PRDM9 to switch to new targets, as observed in many animal taxa.

## Methods

### Data

We used genomic alignments of Chimpanzee (PanTro2 assembly), Denisovan and modern human (hg19 assembly), published by Meyer and colleagues [28] and available at http://cdna.eva.mpg.de/denisova/VCF/hg19_1000g/. Those files contain different information among which we used the following:

human-chimpanzee ancestral sequence inferred by Ensembl Compara EPO 6 primate whole genome alignments (Ensembl release 64) [46]
Denisovan sequence coverage.
A « TS » string indicating the number of different sequences available for each species of the original Ensembl Compara EPO 6 primates whole genome alignment blocks. This field is used to discard paralogous segments.
1000 genomes polymorphism and corresponding averaged allele frequencies (AF) from the 1000 genomes 20101123 intermediate release which contains samples from 1,094 individuals of 15 populations [31].
Duke mappability scores of 20-mers (Map20), which allows the filtering of regions with low mappability quality.
Systematic errors (SysErr), which allows the filtering of regions with low Illumina sequencing quality.
Low Quality (LowQual), which allows to filter regions with uncertain genotype call in Denisovan.

For more information on those annotations, see note 6 in supplementary material of [28].

We used LiftOver software to convert HapMap [2] and DeCODE [35] recombination data from hg18 into hg19 coordinates [47]. This discards 4 HM motifs from experiments using recombination data because their loci are not present in the hg18 assembly.

### Filters

We found 5,704 HM (*CCTCCCTNNCCAC*) and 5,483 CM (*CTTCCCTNNCCAC*) motifs in the human-chimpanzee (HC) reconstructed ancestral sequence described above. Those data were filtered using three increasingly stringent methods named F1, F2 and F3. Each degree of filtering results in a subset of HM and CM motifs (respectively named F1, F2 and F3) used for subsequent analysis. In both motifs the two “N” sites as well as the second position, which is different in CM and HM are classified as non-informative. One motif is used in one given subset if all of its informative sites pass the corresponding level of filtering.

Filter F1 excludes sites that have no genotype call in Denisovan along with those experiencing indels in one of the three species: human, Denisovan or chimpanzee. We excluded indels because the reconstruction of the HC ancestral sequence is particularly difficult at those sites (this specifically excluded 146 HM and 105 CM). This led to the F1 subset composed of 5,474 HM and 5,314 CM motifs.

Filter F2 includes filter F1 and aims at conserving sites for which the HC ancestral sequence is the most reliable. Thus, we only used sites from a filtered subset of the EPO alignment of human, chimpanzee, gorilla and orangutan produced by the Gorilla Sequencing Consortium [48]. This subset has been previously used and filtered as described below and kindly provided by Kasper Munch [40]: “*To increase data quality the alignment is filtered to remove regions of low sequencing quality and regions with a large proportion of gaps or uncalled bases. All alignment blocks that do not contain one and only one sequence for each of the four species are discarded. Then all alignment columns with a gap in both human, chimpanzee and gorilla sequence are removed. To take base call uncertainty into account we then slide a 10nt window by 1nt. If the mean quality score is below 7 the window is removed and the alignment block is split accordingly. To further filter for gap content we slide a window of size 50 by 1nt. If a window contains 49 gaps or more it is removed and the alignment block is split accordingly. Blocks smaller than 300 are removed. The resulting alignment blocks are joined if less than 100 bases apart (and padded accordingly with ‘N’), or split where they contain runs of more than 100 alignment columns of all ‘N’*” [40]. Based on the resulting alignment, we retained only positions for which at least 3 out of the 4 primate species were concordant. This filtered alignment (FA) was used to create the F2 motifs subset: 4,440 HM and 4,393 CM and compute GC* estimates (see below).

Filter F3 includes filter F2 and aims at eliminating potential sequence errors occurring in ancient DNA. This filtering was used in note 9 and following in supplementary material of [28] to estimate substitution rates in Denisovan and *Homo sapiens*. One particular site is excluded if:

it is in a LowQual or a SysErr region as described in the data section above.
it has a Map20 score different from 1 which indicates potential mapping error.
it has a Denisovan sequence coverage below 16 which avoids regions with unreliable Denisovan genotype call.
it has a Denisovan sequence coverage higher than 46 which avoids repeated and duplicated regions.
it is in an EPO alignment block with more than one human sequence or more than one Chimpanzee sequence, which avoids paralogies.

This stringent filtering left only 2,019 HM and 2,274 CM motifs.

The complete list of HM and CM motifs identified is available in the Dataset S2.

### HM and CM motif loss rates estimation

To estimate motif loss rates, the number of motifs that are mutated at informative sites along one branch was counted and then divided by the number of intact motifs found at the ancestral node of this branch. For a given motif, if mutations at informative sites occurred both in the Hominini branch and in one terminal branch (modern human or Denisovan), the motif loss is attributed to the Hominini branch. A mutation is inferred in the chimpanzee branch if the human-chimpanzee ancestral sequence differs from the Chimpanzee sequence. Similarly, a mutation is inferred in the Hominini branch if the human-chimpanzee ancestral sequence differs from the human and Denisovan sequences, with human and Denisovan having the same genotype. Finally, a mutation is inferred in the human (respectively Denisovan) branch if the human-chimpanzee ancestral sequence differs from the human (respectively Denisovan) but not from the Denisovan (respectively human) sequence. If a site is different in the 3 species, it is excluded from the analysis (this concerns only 2 sites excluding 2 distinct HM motifs in the F1 dataset). As the Denisovan genome is diploid, we randomly selected one genotype at heterozygous sites. We repeated all motif loss counts 100 times and provided averaged motif loss rates in Figure 1, Table S1 and S2.

### DAF of mutations affecting motifs in the human branch

To obtain the Derived Allele Frequency (DAF) spectrum of mutations affecting motifs in the human branch, we used the F2 subset of ancestral motifs. We inferred, as previously described, motifs that were present in the human-Denisovan ancestor. For each informative site of those 4,236 HM and 4,218 CM motifs, we computed the DAF using the allele frequency (AF) of the polymorphic sites found in 1000 genomes data if any [31]. If there is no SNP at a particular position (most of them), no DAF is computed except if the reference genome (hg19) is different from the intact motif, in this case this change is considered as fixed (DAF  = 1).

We applied the same approach for chimpanzee, using the polymorphism data derived from the sequencing of the genomes of 10 Western chimpanzees [19] (ftp://birch.well.ox.ac.uk/panMap/haplotypes/VCF/).

### Detection of the THE1 transposable elements

We used the hg19 RepeatMasker 3.3.0 (with repeat library 20120124) list of repeats in the human genome [49] from which we extracted all intervals corresponding to “THE1A”, “THE1A-int”, “THE1B” or “THE1B-int” LTR elements.

### Estimation of dBGC intensity

We fitted a population genetic model to the derived allele frequency (DAF) spectra of CM and HM to estimate the intensity of gene conversion against *PRDM9* motifs, *G* = 4*N_e_g*, using a maximum likelihood framework. We used the model of Eyre-Walker et al. [34] except that we fitted constant positive selection (as gene conversion is equivalent to selection, see [32]) instead of a distribution of deleterious effects. CM sites (resp. HM) play the role of synonymous (resp. non-synonymous) sites. The probability of observing *k_i_* SNPs having *i* derived alleles out of *n* follows a Poisson distribution, *P*(µ,*k_i_*), with mean:
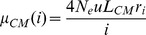
(1a)and
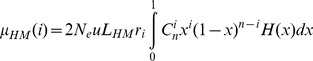
(1b)where *H*(*x*) is the time a converted allele spends between frequency *x* and *x* + *dx*. *N_e_* is the effective population size, *u* the mutation rate, *L_CM_* and *L_HM_* the number of CM and HM motifs, respectively. The *r_i_* have been introduced by Eyre-Walker et al. [34] to take demography and/or population structure (and sampling) into account. There is one *r_i_* for each SNP class, corresponding to the deviation from the standard equilibrium model relative to the singleton class for which *r*_1_ is set to one (for the discussion of the robustness of this kind of model see [34], [50]). The first term within the integral corresponds to the binomial sampling of *i* alleles over *n* given their frequency *x*. Because *n* is very large in the 1000 genomes dataset (*n* = 2,184), we used the continuous approximation that gives very similar results and facilitates numerical computations:



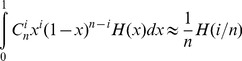
(2)We used the two following nested models:

M0: no conversion
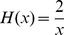
(3)


M1: constant gene conversion of intensity *G* = 4*N_e_g*:
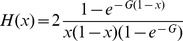
(4)


Because the number of SNPs is much lower than the number of chromosomes sampled, we grouped the SNPs by categories of frequencies. The expectations of these groups of SNPs simply becomes:
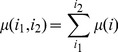
(5)


Assuming independence between motifs, the likelihood of the model can thus be written down as:
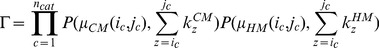
(6)


Parameters estimates were obtained by maximization of the log-likelihood function. The significance of the model with gene conversion is tested by a LRT with 1 degree of freedom. The goodness of fit (*Gof*) of model M1 is assessed by comparing its likelihood with the saturated model for which all *µ* are free. Confidence intervals on *G* are computed by fixing all other parameters at optimum and searching for *G* such that the log-likelihood is two points lower than the maximum likelihood (*lnLmax*).

We tested several values of *m* to assess the robustness of estimations. To do so, we used the following categories of DAF: *f*<0.01 plus *m* other categories defined as 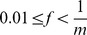 and 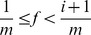 for 1≤*i*≤*m*–1.

Assuming a constant dBGC intensity does not allow to capture the possible very high *G* values in highly recombining regions. To get a better determination of *G*, we assumed that *G* is proportional to recombination rates, *G* =  *c X*, where *X* is the crossover rate (in cM/Mb) in a 2-kb window centered on the motif. We used the observed distribution of *X* in HM motifs. We first fit a gamma distribution to the observed distribution of *X*, which gave a mean of *M_X_*  = 5.02 and a shape of *β* = 0.28. Then we fitted the following model with:
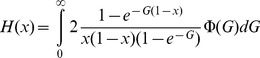
(7)where Φ(G) is a gamma distribution with mean *M_G_*  =  *c M_X_*, and shape *β*, fixed to *β* = 0.28. In this model, *c* is optimized.

### Equilibrium GC content (GC*) estimates

To compute GC* we used the filtered alignment FA corresponding to F2 filter (see above). As GC* estimates are strongly biased by CpG hypermutable sites [51], we discarded all potential CpG sites in the alignment by excluding all G (respectively C) sites for which the previous (respectively following) site is a C (respectively G) in at least one of the four species. We inferred separately AT to GC and GC to AT mutations in each branch of our phylogeny as described above for the count of motif losses. GC* is then computed as follow:
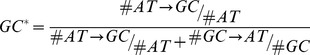
(8)


#X→Y is the number of mutations from X to Y in the branch and #X is the number of X bases in the ancestral node of the branch. “AT” refers either to an “A” or a “T” and “GC” refers either to a “G” or a “C”.

### Statistics

We used normal approximate Z-test with continuity correction to compare motif loss rates. This test is referred as “proportion test” in the text. To compare mean DAFs we used the Wilcoxon test, as DAFs are not distributed normally (Figure 3). All tests and regression computations were made using R software (2.15.0) [52].

## Supporting Information

Figure S1 Derived allele frequency (DAF) spectra of polymorphic mutations leading to motifs loss in the chimpanzee branch. DAF of mutations affecting HM (purple bars) and CM (orange bars) along the chimpanzee branch (black branch in Figure 1). Polymorphism data from [19]. Mutations count for each motif (F2 subset) is indicated (N).
(PDF)

Figure S2 Expected DAF distribution of mutations affecting HM motifs for different dBGC intensities. Derived Allele Frequency (DAF) distribution expected on HM motifs under different dBGC coefficients (G). Equation (4) is plotted for dBGC coefficients ranging from 10 to 1000, as indicated.
(PDF)

Figure S3 Present-day human recombination profiles around HM motifs. DeCODE recombination rates (cM/Mb) around HM motifs found in the human-chimpanzee reconstructed ancestral sequence (Filter F2) and conserved in the human genome (black) or lost in the Hominini (blue) or human (green) branch. The 95% confidence interval of the mean recombination rate is shown by areas colored accordingly. Recombination rates are averaged on 2 kb overlapping windows (overlap  =  1 kb).
(PDF)

Figure S4 Recombination profiles around HM motifs differentiating between fixed and non-fixed losses. DeCODE recombination rates (left panel) and historical recombination rates (right panel) around HM motifs found in the human-chimpanzee reconstructed ancestral sequence (Filter F2) and conserved in the human genome (black) or lost in the human branch (green). If the ancestral allele is present in the 1000 genomes data set [31], the motif loss is considered as not-fixed (dotted line). In the opposite case it is considered as fixed (solid line). The 95% confidence interval of the mean recombination rate is shown by areas colored or hatched accordingly. Recombination rates are averaged on 2 kb overlapping windows (overlap  =  1 kb).
(PDF)

Figure S5 Present-day human recombination profiles around HM motifs lost in the human branch within and outside historical hotspots. DeCODE recombination rates (cM/Mb) around HM motifs found in the human-chimpanzee reconstructed ancestral sequence (Filter F2) within human hotspots (solid line) or outside hotspots (dotted line) and lost in the human branch. The 95% confidence interval of the mean recombination rate is shown by areas colored or hatched accordingly. Recombination rates are averaged on 2 kb overlapping windows (overlap  =  1 kb).
(PDF)

Figure S6 Genome-wide correlations between equilibrium GC content and recombination rate. Each dot represents historical recombination rate (cM/Mb) and equilibrium GC-content (GC*) estimated on the Denisovan branch (red) and human branch (green) over a 1 Mb genomic window.
(PDF)

Figure S7 PRDM9 repeat unit sequences. Zinc finger coding repeat sequences are extracted from [11]. The red horizontal box indicates the 24 bp region used to characterize units in Denisovan sequence data. This region is unique for 10 units out of 20. Blue vertical lines on the left show the 5 pairs of units for which the 24 bp region is identical.
(PDF)

Figure S8 DAF spectra of HM and CM mutations in African (AFR) and European (EUR) populations. Mutations detected along the human branch (N = 594 HM mutations, N = 489 CM mutations; see legend of Figure 3).
(PDF)

Table S1 Motifs loss rates computed on F3 motif subset.
(PDF)

Table S2 Motifs loss rates computed on F1 motif subset.
(PDF)

Table S3 Estimates of the dBGC intensity *G* on HM motifs in the human branch.
(PDF)

Table S4 Estimates of the dBGC intensity G on HM motifs according to local recombination rate.
(PDF)

Table S5 Number of reads matching 24 bp PRDM9 Zn-finger unit specific regions and estimated number of unit copies per genotype in Denisova.
(PDF)

Table S6 Number of W to S substitutions in the Hominini and modern human branches used in Text S3.
(PDF)

Table S7 Number of HM and CM motif losses in the Hominini and modern human branches used in Text S3.
(PDF)

Dataset S1 This PDF file contains sequences of PRDM9 Zn-finger repeat units found in the Denisovan genome: B^den^ and I^den^.
(PDF)

Dataset S2 This archive contains a TAB-delimited data frame with the list of HM and CM motifs found in the HC sequence and a “README” file providing information on this data frame.
(ZIP)

Text S1 Estimating the strength of BGC in favor of hotspot-disrupting alleles.
(PDF)

Text S2 The Denisovan individual PRDM9 does not correspond to any known human allele.
(PDF)

Text S3 Estimation of the onset of human historical hotspots activity along the Hominini branch.
(PDF)
