# Supplementary figures and images for: The Red Queen Model of Recombination Hotspots Evolution in the Light of Archaic and Modern Human Genomes
Source: PLoS Genet. 2014 Nov 13;10(11):e1004790. doi: 10.1371/journal.pgen.1004790 (PMC4230742; doi:10.1371/journal.pgen.1004790)

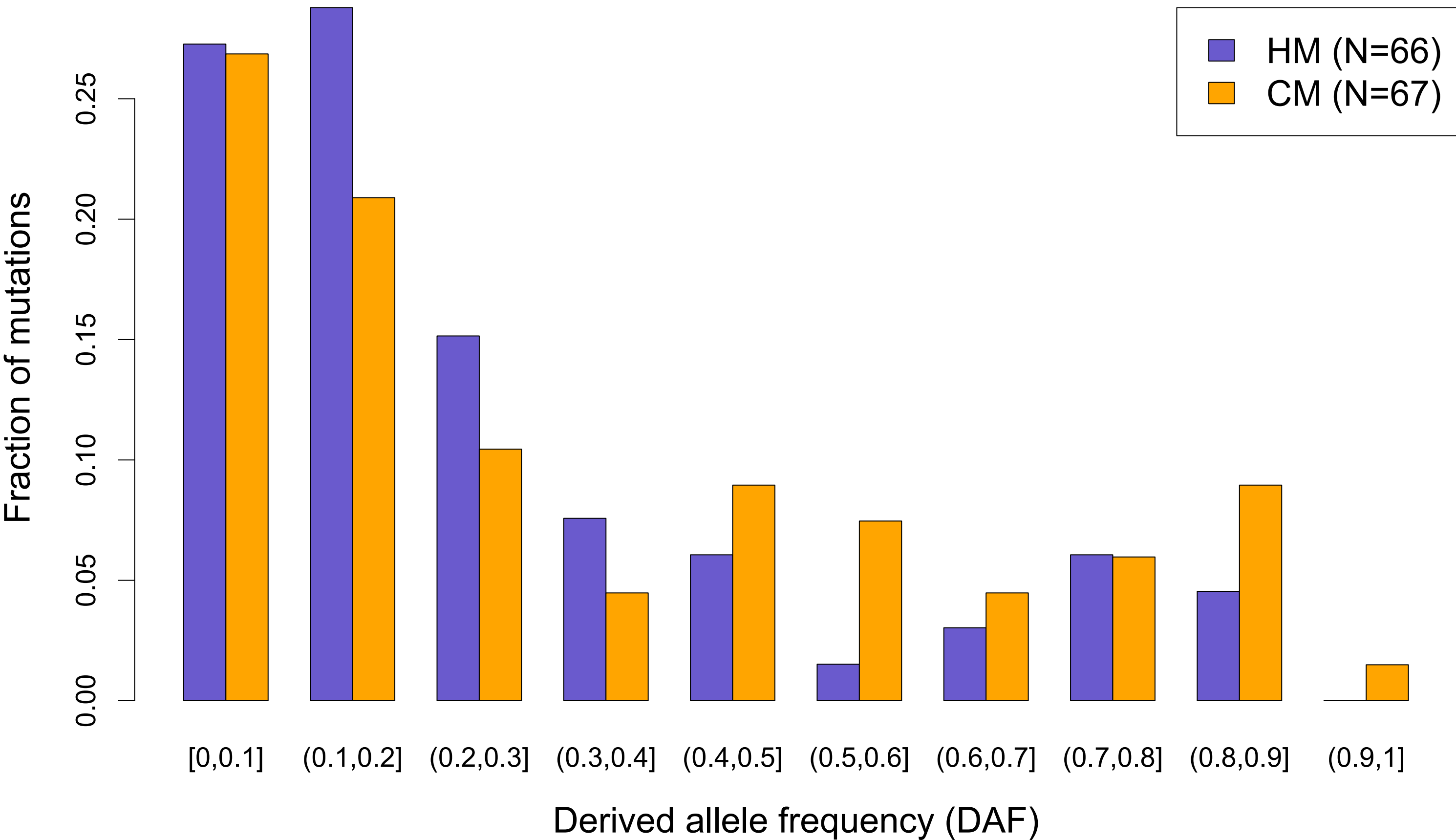

Supplement: Figure S1 — Derived allele frequency (DAF) spectra of polymorphic mutations leading to motifs loss in the chimpanzee branch. DAF of mutations affecting HM (purple bars) and CM (orange bars) along the chimpanzee branch (black branch in Figure 1). Polymorphism data from [19]. Mutations count for each motif (F2 subset) is indicated (N). (PDF) [file pgen.1004790.s001.pdf]

Frequency

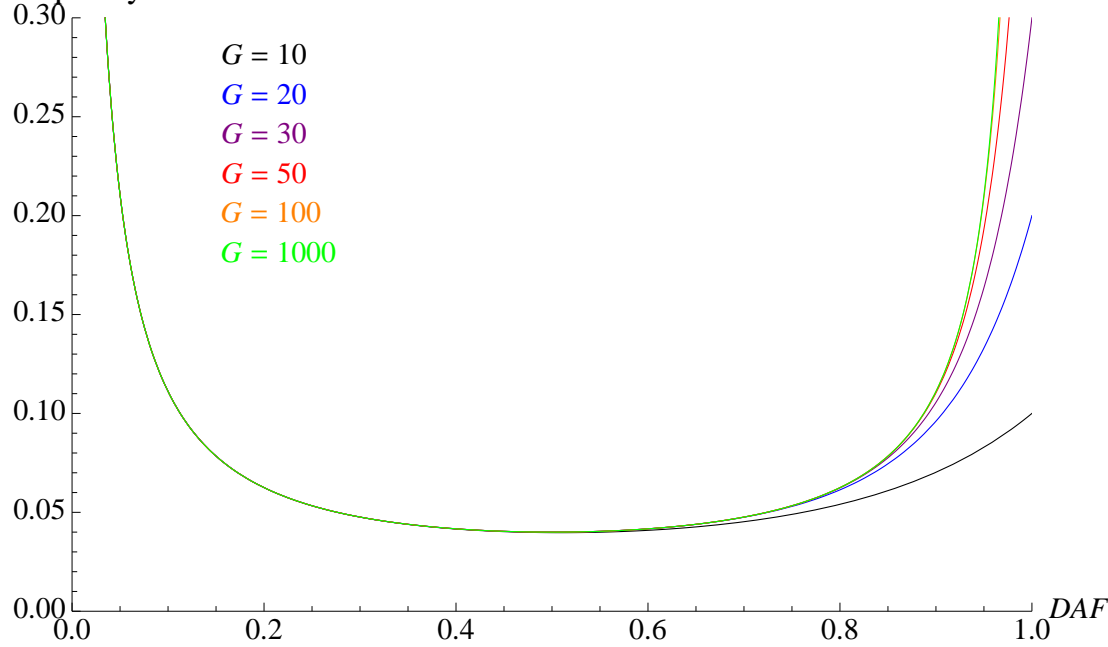

Supplement: Figure S2 — Expected DAF distribution of mutations affecting HM motifs for different dBGC intensities. Derived Allele Frequency (DAF) distribution expected on HM motifs under different dBGC coefficients (G). Equation (4) is plotted for dBGC coefficients ranging from 10 to 1000, as indicated. (PDF) [file pgen.1004790.s002.pdf]

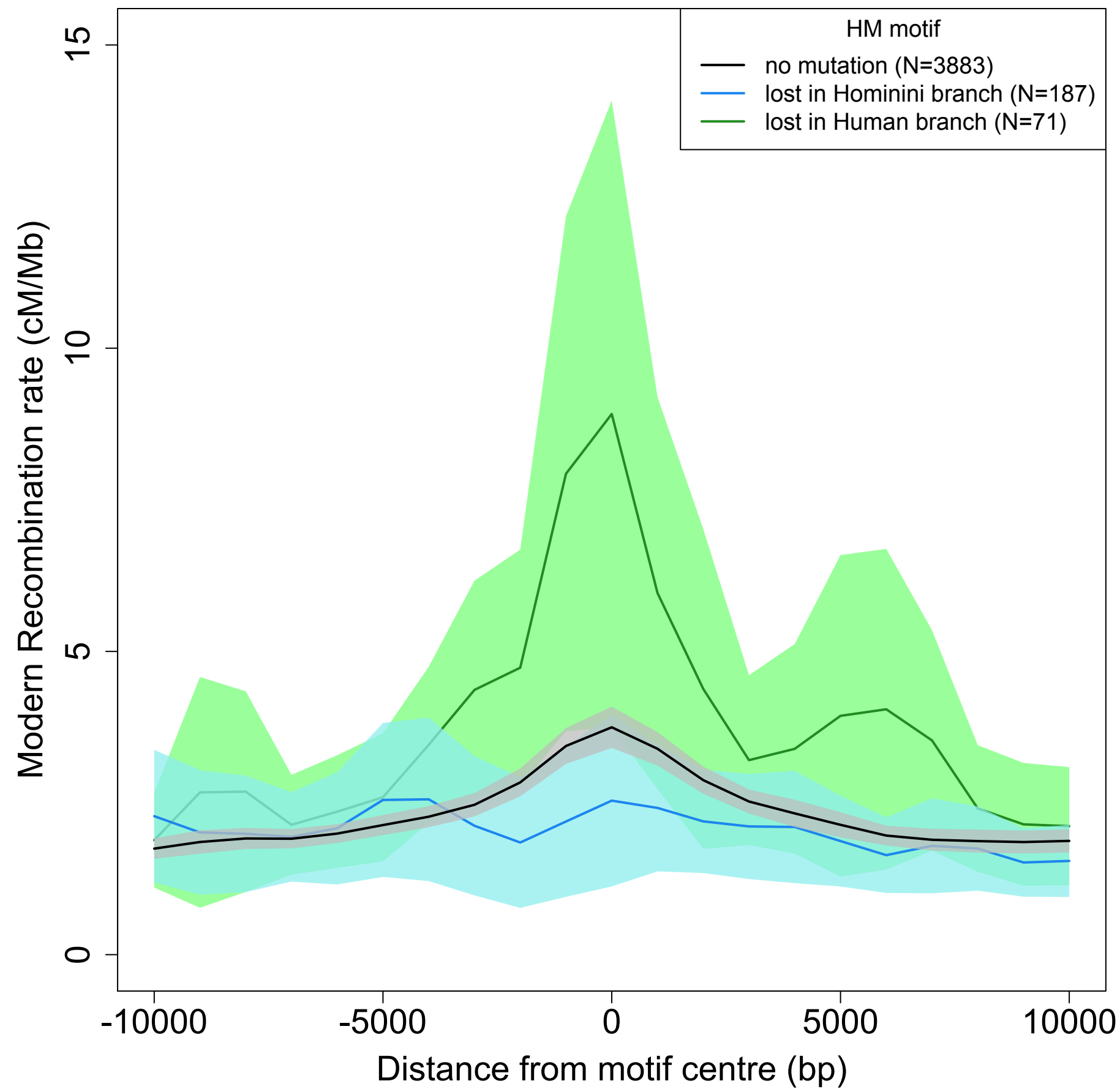

Supplement: Figure S3 — Present-day human recombination profiles around HM motifs. DeCODE recombination rates (cM/Mb) around HM motifs found in the human-chimpanzee reconstructed ancestral sequence (Filter F2) and conserved in the human genome (black) or lost in the Hominini (blue) or human (green) branch. The 95% confidence interval of the mean recombination rate is shown by areas colored accordingly. Recombination rates are averaged on 2 kb overlapping windows (overlap = 1 kb). (PDF) [file pgen.1004790.s003.pdf]

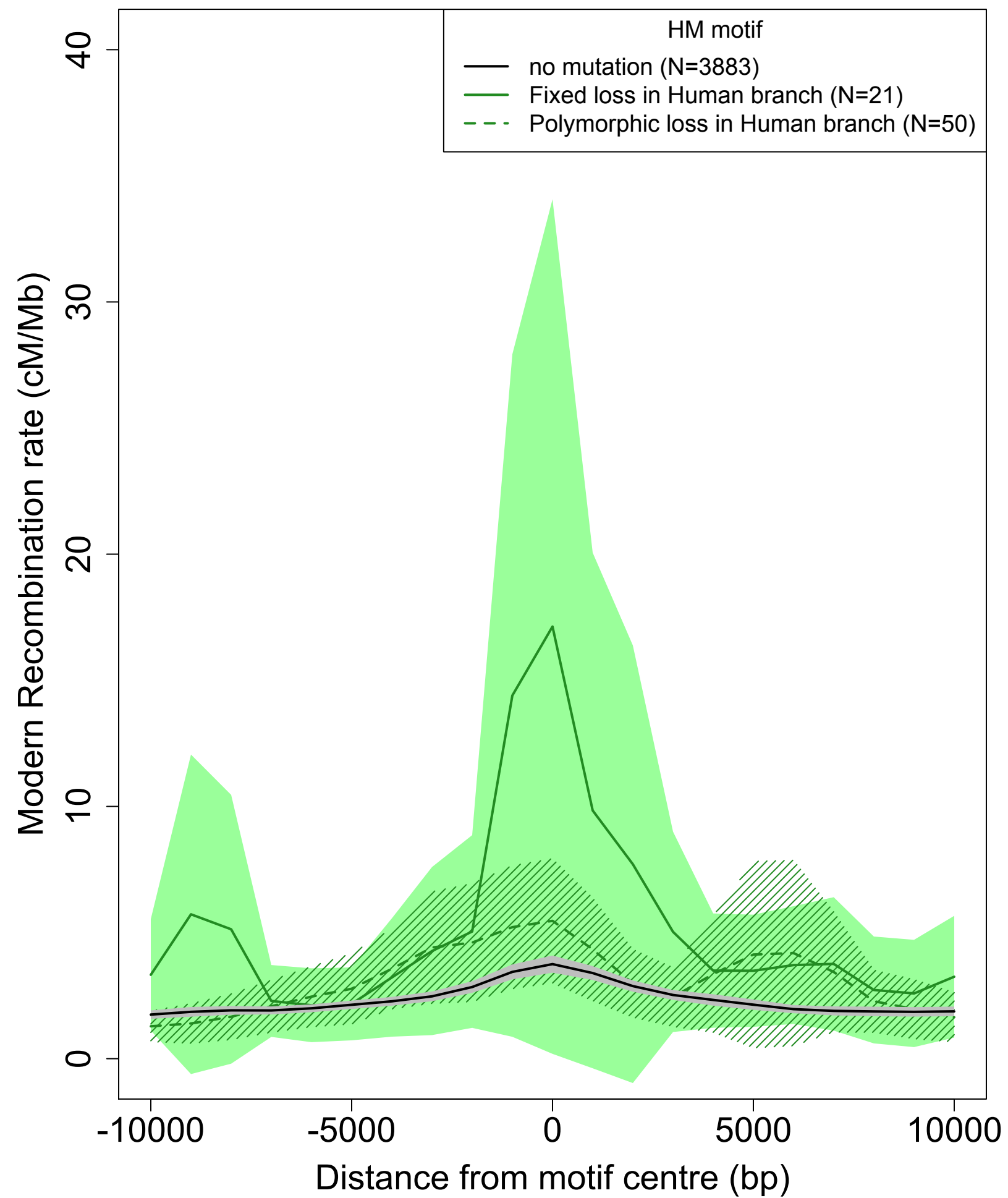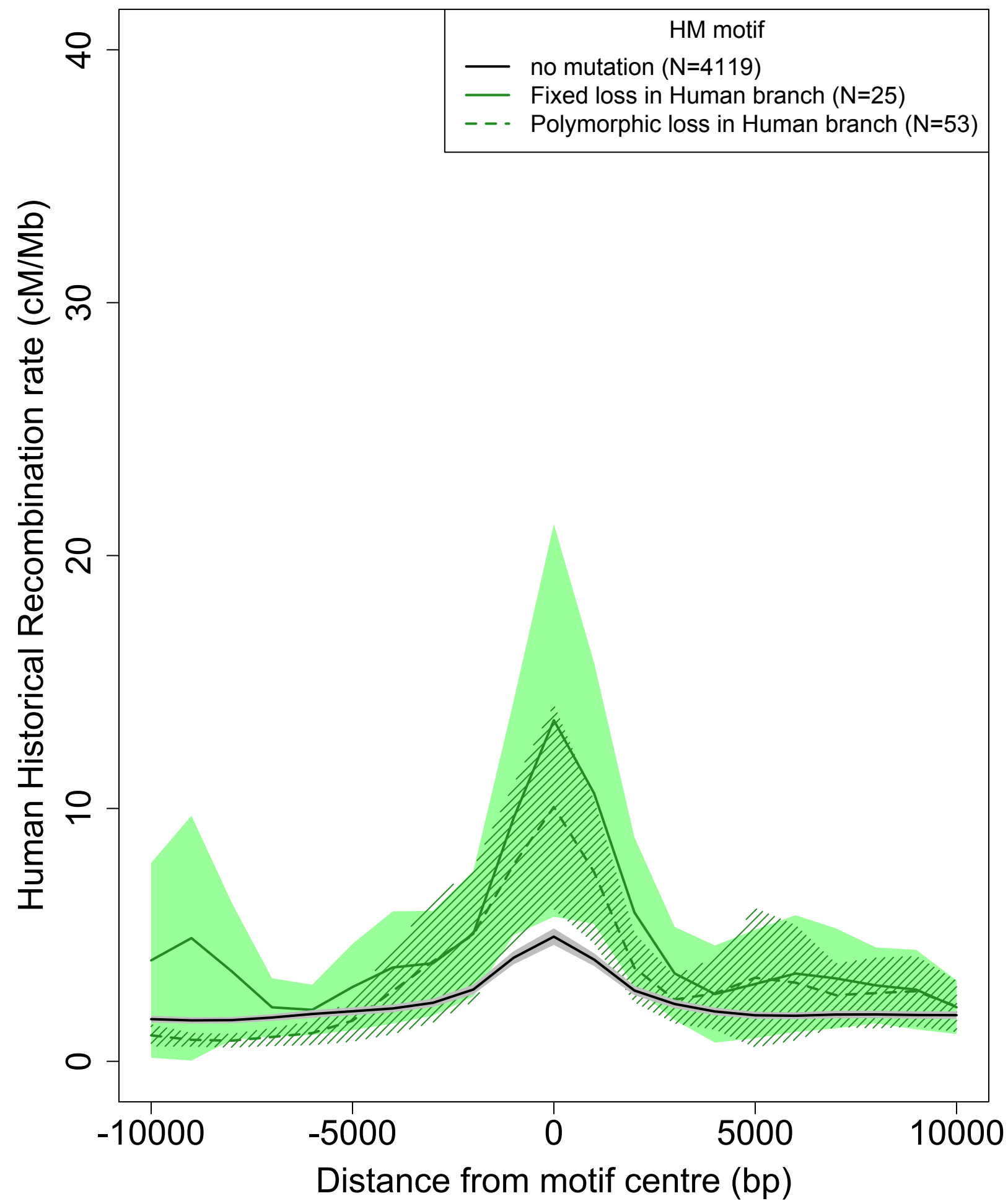

Supplement: Figure S4 — Recombination profiles around HM motifs differentiating between fixed and non-fixed losses. DeCODE recombination rates (left panel) and historical recombination rates (right panel) around HM motifs found in the human-chimpanzee reconstructed ancestral sequence (Filter F2) and conserved in the human genome (black) or lost in the human branch (green). If the ancestral allele is present in the 1000 genomes data set [31], the motif loss is considered as not-fixed (dotted line). In the opposite case it is considered as fixed (solid line). The 95% confidence interval of the mean recombination rate is shown by areas colored or hatched accordingly. Recombination rates are averaged on 2 kb overlapping windows (overlap = 1 kb). (PDF) [file pgen.1004790.s004.pdf]

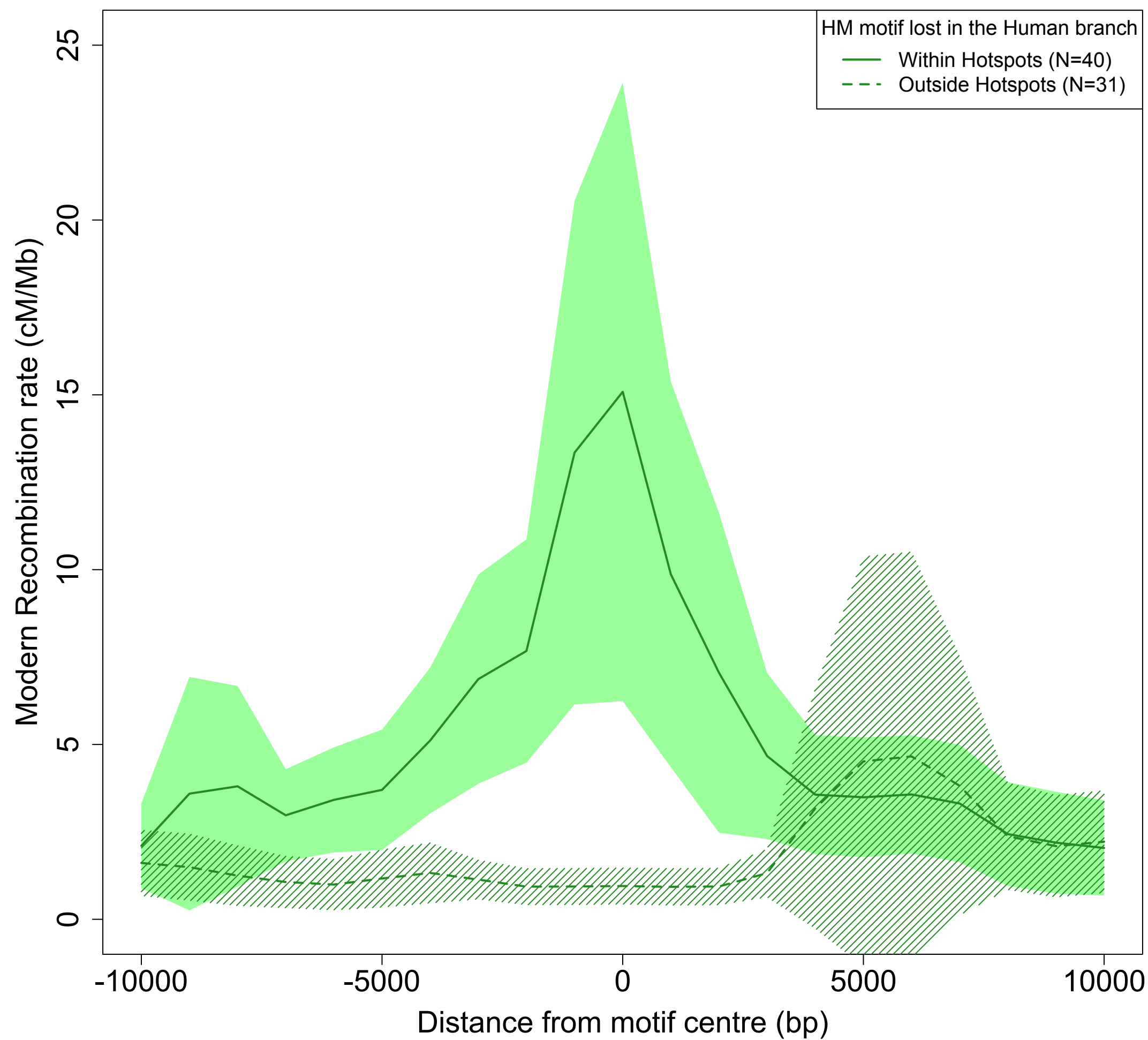

Supplement: Figure S5 — Present-day human recombination profiles around HM motifs lost in the human branch within and outside historical hotspots. DeCODE recombination rates (cM/Mb) around HM motifs found in the human-chimpanzee reconstructed ancestral sequence (Filter F2) within human hotspots (solid line) or outside hotspots (dotted line) and lost in the human branch. The 95% confidence interval of the mean recombination rate is shown by areas colored or hatched accordingly. Recombination rates are averaged on 2 kb overlapping windows (overlap = 1 kb). (PDF) [file pgen.1004790.s005.pdf]

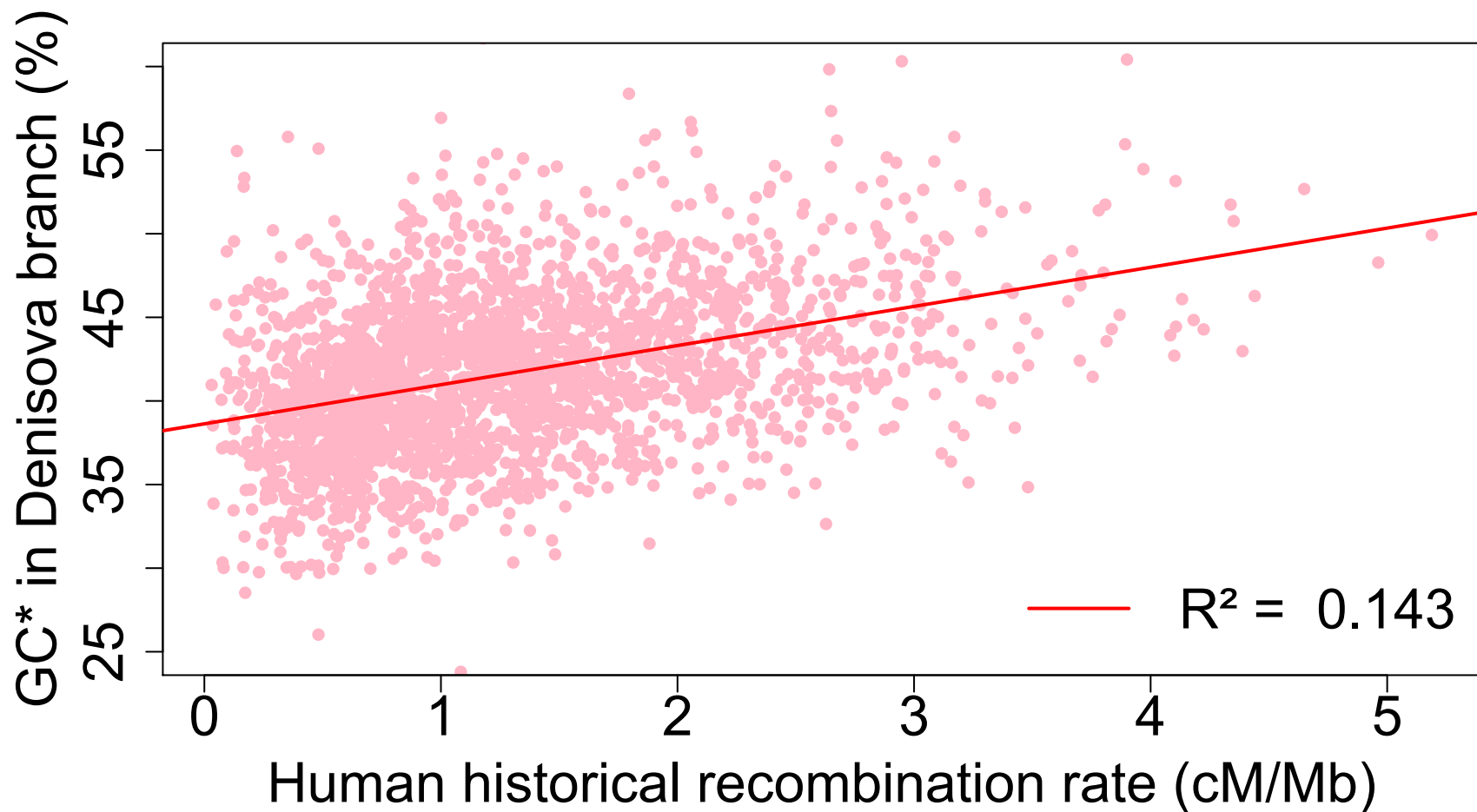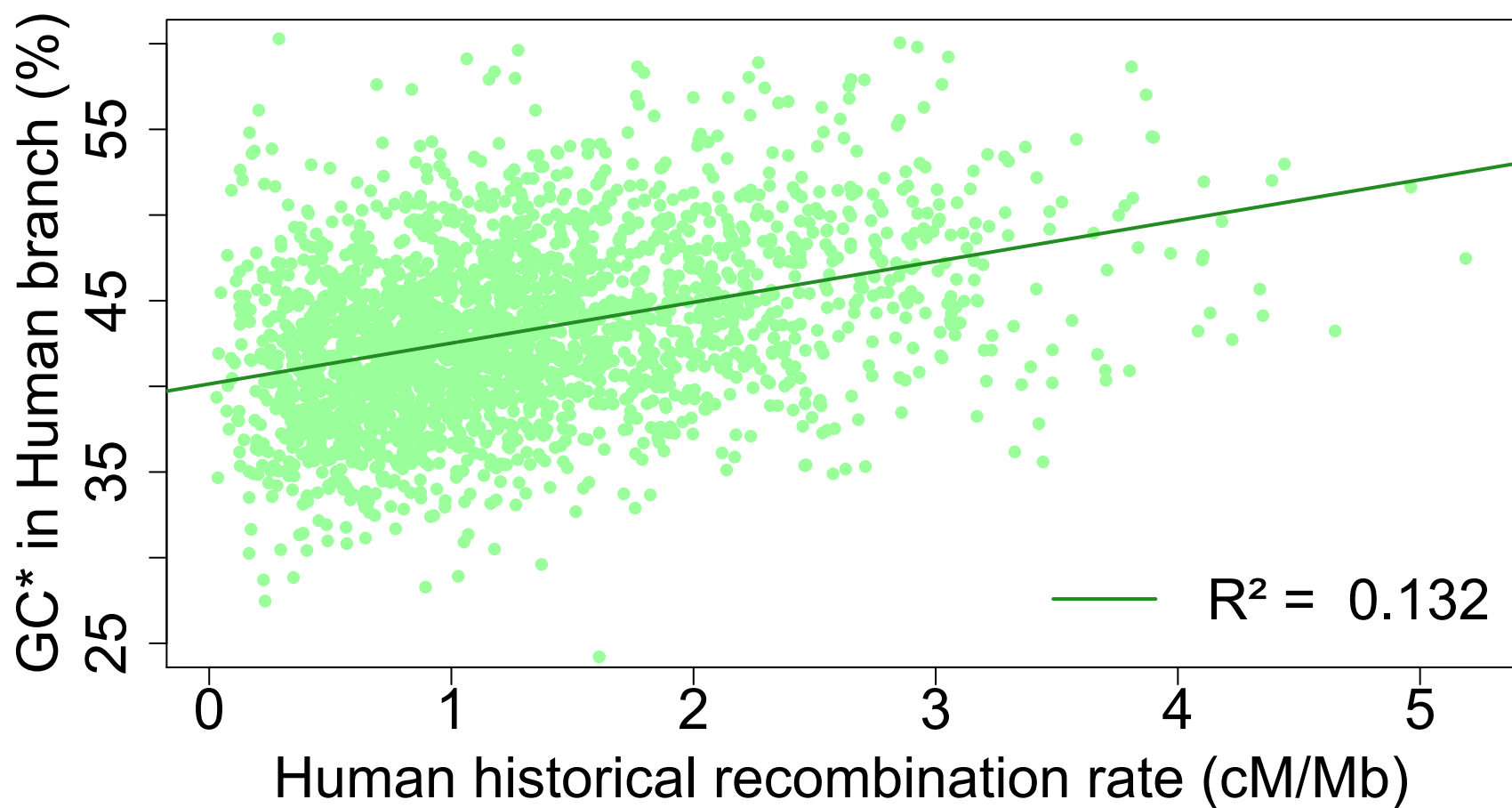

Supplement: Figure S6 — Genome-wide correlations between equilibrium GC content and recombination rate. Each dot represents historical recombination rate (cM/Mb) and equilibrium GC-content (GC*) estimated on the Denisovan branch (red) and human branch (green) over a 1 Mb genomic window. (PDF) [file pgen.1004790.s006.pdf]

Repeat units

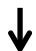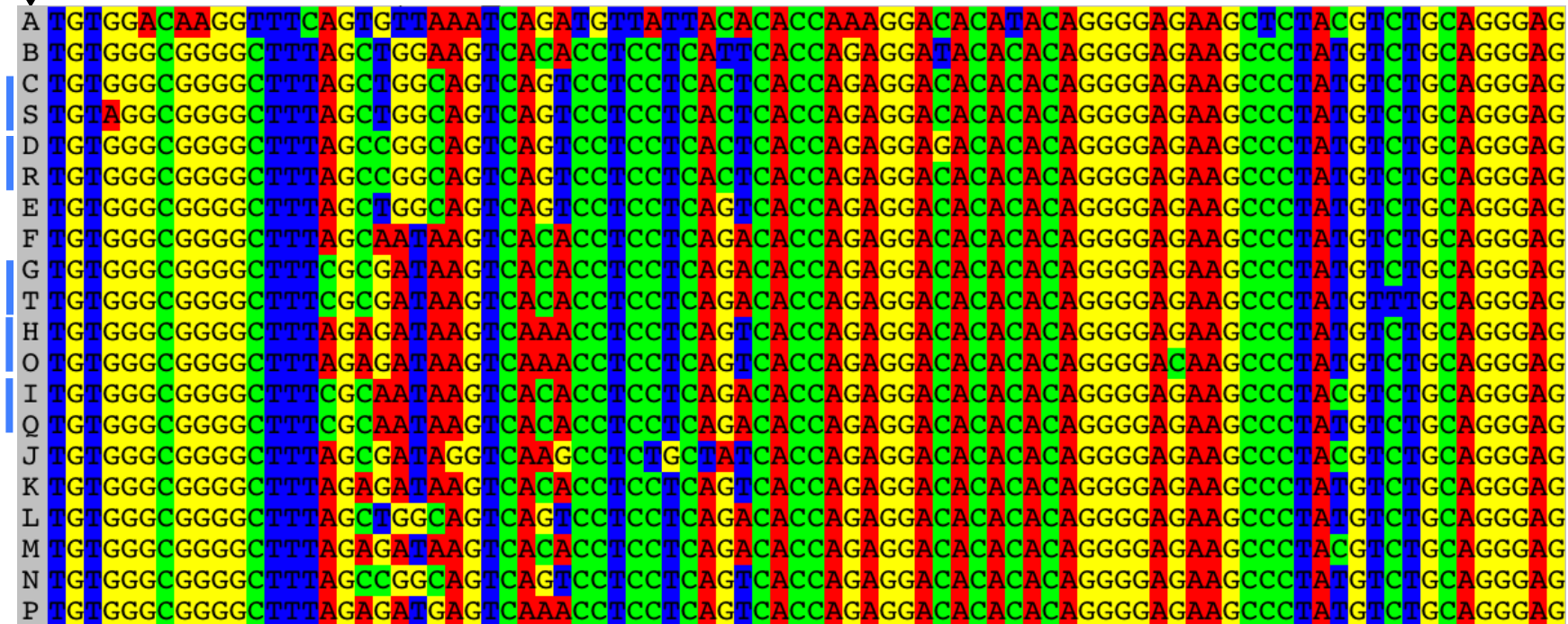

24 bp unit specific region

Supplement: Figure S7 — PRDM9 repeat unit sequences. Zinc finger coding repeat sequences are extracted from [11]. The red horizontal box indicates the 24 bp region used to characterize units in Denisovan sequence data. This region is unique for 10 units out of 20. Blue vertical lines on the left show the 5 pairs of units for which the 24 bp region is identical. (PDF) [file pgen.1004790.s007.pdf]

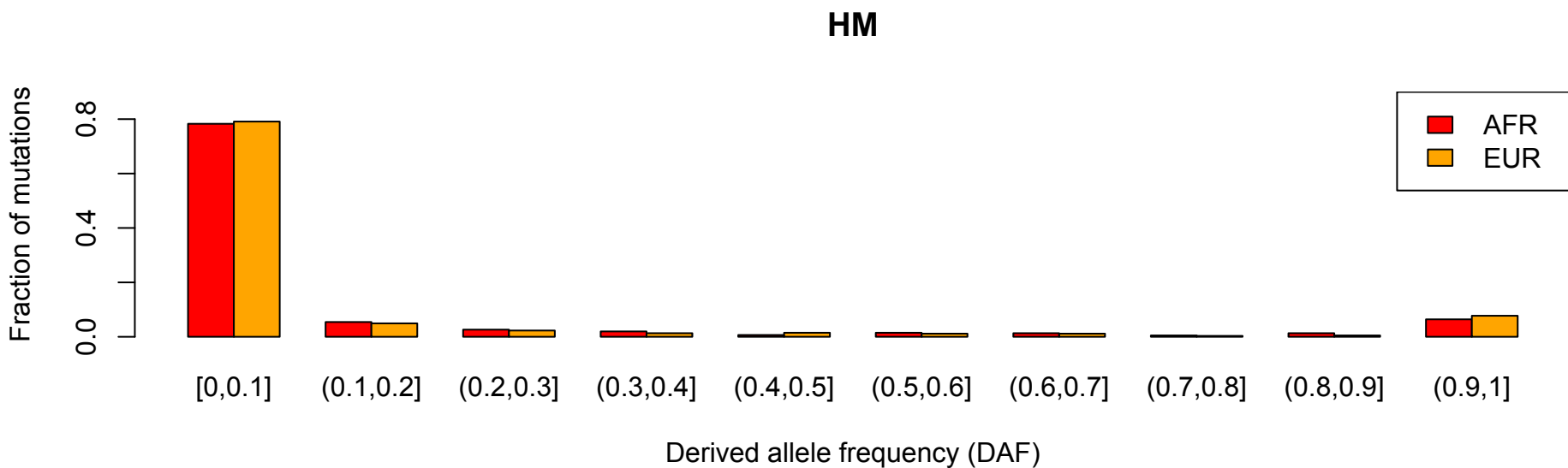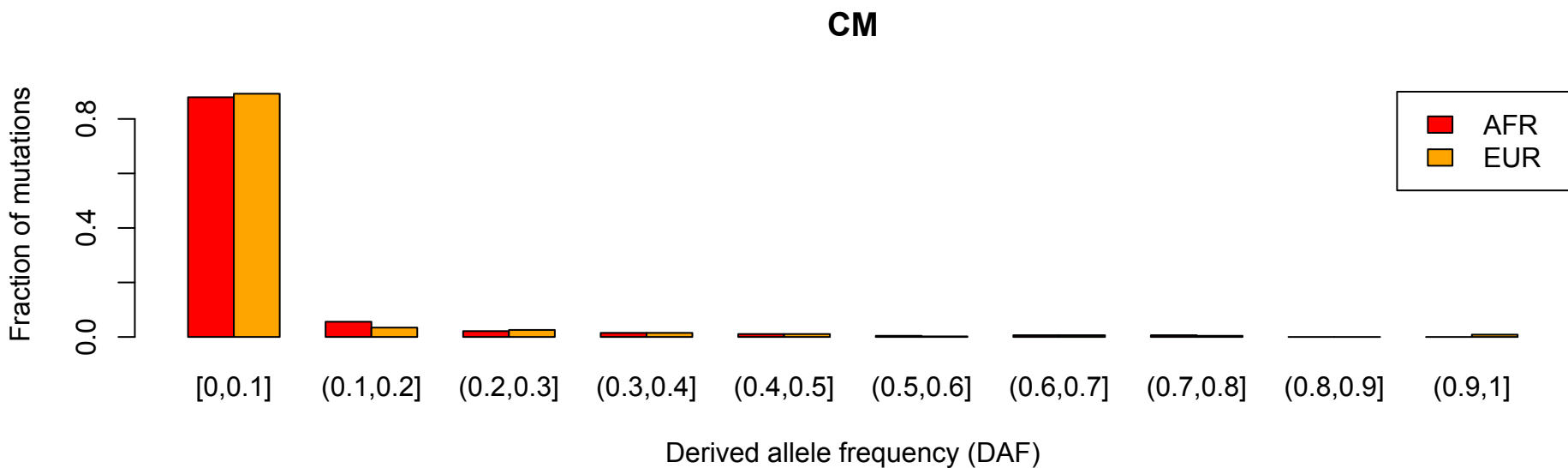

Supplement: Figure S8 — DAF spectra of HM and CM mutations in African (AFR) and European (EUR) populations. Mutations detected along the human branch (N = 594 HM mutations, N = 489 CM mutations; see legend of Figure 3). (PDF) [file pgen.1004790.s008.pdf]
