# Supplementary material for: The Red Queen Model of Recombination Hotspots Evolution in the Light of Archaic and Modern Human Genomes
Source: PLoS Genet. 2014 Nov 13;10(11):e1004790. doi: 10.1371/journal.pgen.1004790 (PMC4230742; doi:10.1371/journal.pgen.1004790)
Supplement: Table S1 — Motifs loss rates computed on F3 motif subset. (PDF) [file pgen.1004790.s009.pdf]

Table S1. Motifs loss rates computed on F3 motif subset.

Branch	N[a]		Rate[b]			p[c]
	HM	CM	HM	CM	HM/CM	
Chimpanzee	2019	2274	5.2%	5.1%	1.0	0.938
Hominini	2019	2274	5.1%	4.3%	1.2	0.071
Denisovan	1908	2177	0.8%	0.4%	2.0	0.078
Human	1908	2177	1.9%	0.4%	4.8	$5.6\ 10^{-6}$

[a] Intact motif count at ancestral node of the branch (cf. Figure 1)

[b] Motif loss rate along the branch

[c] P-value of proportion test comparing HM *vs.* CM loss rates along the branch
