# Supplementary material for: The Red Queen Model of Recombination Hotspots Evolution in the Light of Archaic and Modern Human Genomes
Source: PLoS Genet. 2014 Nov 13;10(11):e1004790. doi: 10.1371/journal.pgen.1004790 (PMC4230742; doi:10.1371/journal.pgen.1004790)
Supplement: Table S2 — Motifs loss rates computed on F1 motif subset. (PDF) [file pgen.1004790.s010.pdf]

Table S2. Motifs loss rates computed on F1 motif subset.

Branch	N[a]		Rate[b]			p[c]
	HM	CM	HM	CM	HM/CM	
Chimpanzee	5474	5314	6.4%	5.8%	1.1	0.209
Hominini	5474	5314	5.3%	4.3%	1.2	0.024
Denisovan	5185	5084	1.1%	0.7%	1.6	0.036
Human	5185	5084	1.8%	0.5%	3.6	$5.7\ 10^{-10}$

[a] Intact motif count at ancestral node of the branch (cf. Figure 1)

[b] Motif loss rate along the branch

[c] P-value of proportion test comparing HM *vs.* CM loss rates along the branch
