# Supplementary material for: The Red Queen Model of Recombination Hotspots Evolution in the Light of Archaic and Modern Human Genomes
Source: PLoS Genet. 2014 Nov 13;10(11):e1004790. doi: 10.1371/journal.pgen.1004790 (PMC4230742; doi:10.1371/journal.pgen.1004790)
Supplement: Table S3 — Estimates of the dBGC intensity G on HM motifs in the human branch. (PDF) [file pgen.1004790.s011.pdf]

Table S3. Estimates of the dBGC intensity *G* on HM motifs in the human branch.

Ncat[a]	G[b]	lnL0[c]	lnL1[c]	lnLmax[d]	p-value[e]	Gof[f]
6	9.30	-43.20	-28.82	-27.91	8.23E-08	0.87
7	7.61	-46.54	-34.26	-31.69	7.14E-07	0.53
10	9.55	-60.29	-46.67	-41.47	1.79E-07	0.32
12	8.55	-67.37	-54.17	-47.35	2.75E-07	0.25
15	6.93	-69.64	-59.81	-55.25	9.27E-06	0.82
22	8.55	-96.06	-82.57	-72.01	2.06E-07	0.45
42	8.26	-137.38	-124.23	-100.97	2.91E-07	0.26

[a] number of DAF categories

[b] Population scaled BGC coefficient ($G = 4N_e g$)

[c] Log-likelihood of neutral model (L0) and BGC model (L1)

[d] see methods

[e] p-value of LRT test comparing M0 and M1

[f] Goodness of fit (see methods)
