# Supplementary material for: The Red Queen Model of Recombination Hotspots Evolution in the Light of Archaic and Modern Human Genomes
Source: PLoS Genet. 2014 Nov 13;10(11):e1004790. doi: 10.1371/journal.pgen.1004790 (PMC4230742; doi:10.1371/journal.pgen.1004790)
Supplement: Table S4 — Estimates of the dBGC intensity G on HM motifs according to local recombination rate. (PDF) [file pgen.1004790.s012.pdf]

Table S4. Estimates of the dBGC intensity *G* on HM motifs according to local recombination rate.

Rate[a]	CM[b]	HM[b]	G[c]	lnL0[d]	lnL1[d]	lnLmax[e]	p-value[f]	Gof[g]
0.098	1736	1177	0.96	-38.11	-37.94	-28.85	0.565	0.052
0.565	1562	1407	4.24	-40.47	-38.45	-34.90	0.045	0.716
9.412	1092	1852	14.64	-49.10	-40.66	-34.97	3.96E-05	0.330

[a] Mean human historical recombination rate (cM/Mb) over 2 kb window around motifs

[b] Number of CM and HM motifs used in this recombination category

[c] Population scaled BGC coefficient ($G = 4N_e g$)

[d] Log-likelihood of neutral model (L0) and BGC model (L1)

[e] see methods

[f] p-value of LRT test comparing M0 and M1

[g] Goodness of fit (see methods)
