# Supplementary material for: The Red Queen Model of Recombination Hotspots Evolution in the Light of Archaic and Modern Human Genomes
Source: PLoS Genet. 2014 Nov 13;10(11):e1004790. doi: 10.1371/journal.pgen.1004790 (PMC4230742; doi:10.1371/journal.pgen.1004790)
Supplement: Table S5 — Number of reads matching 24 bp PRDM9 Zn-finger unit specific regions and estimated number of unit copies per genotype in Denisova. (PDF) [file pgen.1004790.s013.pdf]

Table S5. Number of reads matching 24 bp PRDM9 Zn-finger unit specific regions and estimated number of unit copies per genotype in Denisova.

Unit region	A	B*	C/S	D/R	E	F	G/T	H/O	I*/Q	J	K	L	M	N	P
R[a]	16	20	62	62	0	42	0	60	20	17	0	0	0	0	0
N[b]	2	2|3	7|8	7|8	0	5|6	0	7|8	2|3	2|3	0	0	0	0	0

[a] Number of reads matching the 24 bp region

[b] Estimated number of copies of the unit per genotype. "2|3" stands for "2 or 3 copies per genotype".

* reads are from B^{den} and I^{den} units
