# Supplementary material for: The Red Queen Model of Recombination Hotspots Evolution in the Light of Archaic and Modern Human Genomes
Source: PLoS Genet. 2014 Nov 13;10(11):e1004790. doi: 10.1371/journal.pgen.1004790 (PMC4230742; doi:10.1371/journal.pgen.1004790)
Supplement: Table S6 — Number of W to S substitutions in the Hominini and modern human branches used in Text S3. (PDF) [file pgen.1004790.s014.pdf]

Table S6. Number of W to S substitutions in the Hominini and modern human branches used in Text S3.

	Focal sites (F)	Background sites (B)
Number of W sites in the human-chimpanzee ancestor	F_a = 1699374	B_a = 1726440
Number of W to S substitutions in the Hominini branch	F_{12} = 7097	B_{12} = 6742
Number of W to S substitutions in the modern human branch	F_3 = 207	B_3 = 83
