# Supplementary material for: The Red Queen Model of Recombination Hotspots Evolution in the Light of Archaic and Modern Human Genomes
Source: PLoS Genet. 2014 Nov 13;10(11):e1004790. doi: 10.1371/journal.pgen.1004790 (PMC4230742; doi:10.1371/journal.pgen.1004790)
Supplement: Table S7 — Number of HM and CM motif losses in the Hominini and modern human branches used in Text S3. (PDF) [file pgen.1004790.s015.pdf]

Table S7. Number of HM and CM motif losses in the Hominini and modern human branches used in Text S3.

	HM motifs (F)	CM motifs (B)
Number of motifs in the human-chimpanzee ancestor	F_a = 4440	B_a = 4392
Number of motif losses in the Hominini branch	F_{12} = 204	B_{12} = 179
Number of motif losses in the modern human branch	F_3 = 25	B_3 = 1
