# Supplementary material for: The Red Queen Model of Recombination Hotspots Evolution in the Light of Archaic and Modern Human Genomes
Source: PLoS Genet. 2014 Nov 13;10(11):e1004790. doi: 10.1371/journal.pgen.1004790 (PMC4230742; doi:10.1371/journal.pgen.1004790)
Supplement: Dataset S1 — This PDF file contains sequences of PRDM9 Zn-finger repeat units found in the Denisovan genome: Bden and Iden. (PDF) [file pgen.1004790.s016.pdf]

>B_den
TGTGGGCGGGGCTTTAGCTGGAAGTCACACCTCCTCATTCACCAGAGGATACACACAGGAGAGAAGCCCTATGTCTGCAG
GGAG

>I_den
TGTGGGCGGGGTTTTCGCAATAAGTCACACCTCCTCAGACACCAGAGGACACACACAGGGGAGAAGCCCTACGTCTGCAG
GGAG
