# Supplementary material for: The Red Queen Model of Recombination Hotspots Evolution in the Light of Archaic and Modern Human Genomes
Source: PLoS Genet. 2014 Nov 13;10(11):e1004790. doi: 10.1371/journal.pgen.1004790 (PMC4230742; doi:10.1371/journal.pgen.1004790)
Supplement: Text S1 — Estimating the strength of BGC in favor of hotspot-disrupting alleles. (PDF) [file pgen.1004790.s018.pdf]

Supplementary Text S1: Estimating the strength of BGC in favor of hotspot-disrupting alleles

To estimate the intensity of BGC against HM motifs, we analyzed the DAF spectra of CM and HM mutations (see main text). To take into account the variability in local recombination rates, we considered a simple model, where the population-scale BGC parameter (G = $4N_eg$) at a given HM motif is directly proportional to the local crossover rate at this locus ($G = c X$; where X is the crossover rate in a 2-kb window centered on the motif, in cM/Mb). We estimated the value of c by fitting a population genetic model to the DAF spectra of CM and HM mutations. This procedure gives a ML estimate of c = 4.8 (CI: 0.8 - 125000). The upper bound of this confidence interval is extremely high. This is due to the fact that DAF spectra do not allow one to differentiate between strong values of G.

To obtain a more realistic upper bound for the estimate of c, we used the model developed by [1], which describes BGC in favor of hotspot-disrupting alleles. Let us consider a window of 2-kb centered on a HM motif, with two alleles: A corresponding to the intact HM motif, and B to the mutated motif. In an AB heterozygote, a chromosome with the A allele initiates a DSB within that window with probability r_A and a chromosome with the B allele initiates a DSB in this window with probability r_B (with $r_A > r_B$). When a DSB is initiated then with probability p, the allele that initiated the DSB is transmitted. Biologically possible values of p range from p=0 (the DSB-carrying allele is systematically converted by the other one) to p=1/2 (no transmission bias). According to this model, the population-scale BGC parameter (G = $4N_eg$, where N_e is the effective population size and g the BGC coefficient) is given by:

$$G = 8N_e(r_A - r_B)(\frac{1}{2} - p) \quad (1)$$

It is important to note that the repair of DSB can lead either to crossover (CO) or non-crossover (NCO) recombination events. Thus, the frequency of DSB formation in the window is distinct from the frequency of crossovers. G can be expressed according to the local crossover rate (X, in cM/Mb), with the formula:

$$G = 8N_e\left(\frac{X_A - X_B}{f}\right)\left(\frac{1}{2} - p\right)\frac{2000}{100\times10^6} = 1.6\ 10^{-4}N_e\left(\frac{X_A - X_B}{f}\right)\left(\frac{1}{2} - p\right) \quad (2)$$

where f is the fraction of DSBs that are repaired as crossover events.

In theory, the maximum possible value of G could be obtained for p=0 (if all DSBs initiated within the window occurred very close to the HM motif) and r_B=0 (if the mutation of the motif totally prevented the formation of DSBs in the window):

$$G_{max} = 8\ 10^{-5}N_e\left(\frac{X_A}{f}\right) \quad (3)$$

Empirical data indicate that mutations of the HM motif generally do not abolish the activity of hotspots (see main text). Thus, in reality r_B>0, and hence G_{max} corresponds to an upper bound for the true value of G.

The level of polymorphism in human populations corresponds to an effective population size of about 10,000. The total number of DSBs per genome is about 10 times the number of crossover [2]. With these parameters (f = 0.1 and N_e =10,000), this would give:

$$G_{max} = 8\,X_A \qquad (4)$$

Hence, this leads to a more realistic upper bound for estimate of c = 8.

References

1. Coop G, Myers SR (2007) Live hot, die young: transmission distortion in recombination hotspots. PLoS Genet 3: e35. doi:10.1371/journal.pgen.0030035.

2. Baudat F, Imai Y, de Massy B (2013) Meiotic recombination in mammals: localization and regulation. Nat Rev Genet 14: 794–806. doi:10.1038/nrg3573.
