# Supplementary material for: The Red Queen Model of Recombination Hotspots Evolution in the Light of Archaic and Modern Human Genomes
Source: PLoS Genet. 2014 Nov 13;10(11):e1004790. doi: 10.1371/journal.pgen.1004790 (PMC4230742; doi:10.1371/journal.pgen.1004790)
Supplement: Text S2 — The Denisovan individual PRDM9 does not correspond to any known human allele. (PDF) [file pgen.1004790.s019.pdf]

Supplementary Text S2: The Denisovan individual PRDM9 does not correspond to any known human allele

In humans, 29 different alleles of PRDM9 have been described (named from A to E and L1 to L24) [1]. These alleles differ by the number (8 to 18), the identity and the order of minisatellite repeat units (84 bp long) that encode the Zn-finger DNA-binding domain (named from A to T). It is impossible to assemble properly the sequence of this minisatellite repeat in Denisovan, because ancient DNA is highly fragmented (the median size of sequence reads in the Denisovan data set is 56 bp). However, we can use two types of information from reads mapped at the PRDM9 locus to get insight on the alleles present in the Denisovan individual.

First, reads mapped on the human PRDM9 locus display two synonymous SNPs. Those are respectively located in the B and I repeat units of the reference genome. At those sites (chr5: 23,526,925 and 23,527,717 respectively) all mapped Denisovan reads are mutated (G->A and C->T respectively) meaning that units B and I, as known in present human populations, are absent from the ancient genome. Additionally, both of those mutations do not correspond to any other PRDM9 repeat unit known so far in humans. Hereafter, we call the new corresponding repeat units B^{den} and I^{den} (see supplementary data). Thus, PRDM9 Denisovan individual genotype does not correspond to any human PRDM9 genotype described so far. However, as those changes are synonymous, B^{den} and I^{den} are likely to be functionally identical to units B and I respectively. Thus this analysis does not provide information on the PRDM9 target motif recognized by the corresponding alleles (i.e. the PRDM9 phenotype).

Secondly, the analysis of sequence coverage allowed us to estimate the copy number of each of the different units known to constitute the Zn-finger domain of the Denisovan individual. To characterize repeat units, we used only the 24 bp variable region of those units (positions 16 to 39; Figure S7). For 10 characterized units out of 20 (A, B, E, F, J, K, L, M, N and P) this region is unique among all described units. For the 10 others, the sequence of this region is shared by one and only one other unit among the 20: C/S, D/R, G/T, H/O and I/Q (Figure S7). NB: B^{den} and I^{den} units have the same 24 bp region than units B and I/Q respectively. Then we extracted a wide set of mapped Denisovan reads potentially resulting from the sequencing of the PRDM9 Zn-finger locus. This includes the PRDM9 Zn-finger region ±100 bp (chr5: 23,526,242 - 23,528,806; 1,344 reads) and the homologous region of the PRDM7 locus (chr16: 90,123,447 - 90,125,042; 869 reads), which is a close paralog to PRDM9. For each of the 15 specific 24 bp regions (A, B, E, F, J, K, L, M, N, P, C/S, D/R, G/T, H/O and I/Q), we counted the number of reads containing a segment with 100% identity with the region (Table S5). Noting that all PRDM9 alleles known so far have one and only one A unit, we can consider that the number of reads matching the 24 bp region of unit A reflects the coverage of a unit represented once by allele (twice per diploid genotype). Assuming that the number of copies is proportional to the corresponding coverage of the 24 bp specific region of this unit, we estimated copy number of each repeat unit per genotype (Table S5). The pattern of estimated copy number found in Denisovan is not compatible with any combination (homozygous and heterozygous) of alleles known so far in humans.

Reference

1. Berg IL, Neumann R, Lam K (2010) PRDM9 variation strongly influences recombination hot-spot activity and meiotic instability in humans. Nature 42: 859–863. doi:10.1038/ng.658.PRDM9.
