# Supplementary material for: The Red Queen Model of Recombination Hotspots Evolution in the Light of Archaic and Modern Human Genomes
Source: PLoS Genet. 2014 Nov 13;10(11):e1004790. doi: 10.1371/journal.pgen.1004790 (PMC4230742; doi:10.1371/journal.pgen.1004790)
Supplement: Text S3 — Estimation of the onset of human historical hotspots activity along the Hominini branch. (PDF) [file pgen.1004790.s020.pdf]

Supplementary Text S3: Estimation of the onset of human historical hotspots activity along the Hominini branch.

1) Principle of the approach

To date when human historical hotspots started to be active, we searched for two signatures of recombination activity: BGC against PRDM9 target motifs, and BGC in favor of GC-alleles (gBGC). The first type of BGC can be detected by comparing the loss rate of HM motifs to the loss rate of CM motifs (see main text). The process of gBGC can be detected by analyzing substitution patterns, to infer the equilibrium GC-content (denoted GC*). Along the human branch, as expected, there is a strong signal for these two signatures (see main text). Interestingly, along the Hominini branch, we observed a small bump of GC* in the center of loci corresponding to human historical recombination hotspots (Figure 6B). This indicates that these historical hotspots (or at least a fraction of them) were already active before the population split between Denisovans and modern humans. Moreover, we also observed a slight excess of HM losses over CM losses along the Hominini branch. This suggests that HM started to be a target of PRDM9 before this split.

To try to date the onset of activity of human historical recombination hotspots, we considered a simple model, assuming that all human recombination hotspots started to be active at the same date and that their intensity has been constant since then. Thus, according to this model, loci corresponding to human historical recombination hotspots have been subject to constant BGC during the time period t_2+t_3, but were not affected by BGC during the time period t_1:

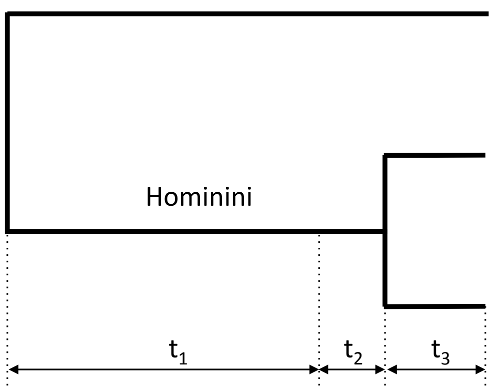

Our goal here is to estimate t_2. Given the uncertainties about the human-chimpanzee divergence time [1], we will express the date of the onset of recombination hotspots as a fraction (*f*) of the Hominini branch:

$f = \frac{t_2}{t_1+t_2}$: fraction of the Hominini branch during which hotspots have been active.

Let us consider two categories of sites: focal sites (F), subject to BGC during the time period t_2+t_3, and background sites (B) not affected by BGC. We will use the following notation:

u : substitution rate (per site per unit of time) in absence of BGC

v : substitution rate (per site per unit of time) at sites affected by BGC

F_a : number of focal sites at the root of the tree

F_{12} : total number of substitutions at focal sites during time t_1+t_2

F_3 : total number of substitutions in the human branch at focal sites during time t_3

B_a : number of background sites at the root of the tree

B_{12} : total number of substitutions at background sites during time t_1+t_2

B_3 : total number of substitutions in the human branch at background sites during time t_3

Given the very short evolutionary distances considered here, the number of substitutions along the Hominini branch can be approximated by:

$$B_{12} = B_a\, u\, (t_1 + t_2) \qquad (1)$$

$$F_{12} = F_a\, (u\, t_1 + v\, t_2) \qquad (2)$$

From this, one can obtain:

$$\frac{F_{12}}{B_{12}} = \frac{F_a}{B_a}\left(1 + f\left(\frac{v}{u} - 1\right)\right) \qquad (3)$$

and hence:

$$f = \left(\frac{F_{12}B_a}{B_{12}F_a} - 1\right)\left(\frac{v}{u} - 1\right)^{-1} \qquad (4)$$

The number of substitutions along the human branch can be expressed as:

$$B_3 = (B_a - B_{12})\, u\, t_3 \qquad (5)$$

$$F_3 = (F_a - F_{12})\, v\, t_3 \qquad (6)$$

From this, one can obtain the ratio v/u:

$$\frac{v}{u} = \left(\frac{F_3}{F_a - F_{12}}\right)\left(\frac{B_a - B_{12}}{B_3}\right) \qquad (7)$$

And hence, from equation (4):

$$f = \left(\frac{F_{12}B_a}{B_{12}F_a} - 1\right)\frac{(F_a - F_{12})B_3}{(B_a - B_{12})F_3 - (F_a - F_{12})B_3} \qquad (8)$$

2) Dating the onset of gBGC activity at human historical hotspots

To date the onset of gBGC activity, we computed the number of W (A or T) to S (G or C) substitutions (using the F2 sequence alignment data set) along the different branches of the phylogeny, in a window of 100 bp centered on the middle of each of the 32,981 human historical hotspots (focal sites). As a reference of sites not affected by gBGC (background sites), we

considered two 50 bp-long windows, located respectively 10 kb upstream and downstream of the center of human historical hotspots. To determine whether mutations along the modern human branch were fixed (i.e. substitutions) or not, we used polymorphism data from the 1000 Genomes project (dataset 20101123 intermediate release).

Taking substitution counts from Table S6, and using equation (8) we inferred f = 4.5%.

The human-chimpanzee divergence time is estimated to 7-13 MYR ago (Langergraber et al. 2012). The human-Denisovan population split is estimated to 0.4-0.8 MYR ago (Langergraber et al. 2012). Thus, the onset of human historical hotspot activity is dated to 0.7 MYR ago (if we consider the lower estimates of divergence times) or 1.3 MYR ago (if we consider the upper estimates of divergence times).

3) Dating the onset of BGC activity on HM motifs

To date the onset of BGC on PRDM9 motifs, we computed the number of HM motif losses (focal sites) fixed along the different branches of the phylogeny (using the F2 sequence alignment data set). We used CM motifs as a reference of sites not affected by BGC (background sites). To determine whether mutations along the modern human branch were fixed (i.e. substitutions) or not, we used polymorphism data from the 1000 Genomes project (dataset 20101123 intermediate release).

Taking substitution counts from Table S7, and using equation (8) we inferred f = 0.5%.

Thus, this analysis suggests that the HM motif started to be the target of PRDM9 0.44 MYR ago (if we consider the lower estimates of divergence times) or 0.87 MYR ago (if we consider the upper estimates of divergence times).

Given the limited number of observed motif losses (notably in the modern human branch), these values have to be considered as very rough estimates. However, this result is in agreement with the analyses of gBGC signatures, which indicate that human historical hotspots started to be active shortly before the human-Denisovan population split.

<u>Reference</u>

1. Langergraber KE, Prüfer K, Rowney C, Boesch C, Crockford C, et al. (2012) Generation times in wild chimpanzees and gorillas suggest earlier divergence times in great ape and human evolution. Proc Natl Acad Sci U S A 109: 15716–15721. doi:10.1073/pnas.1211740109.
